# Mesenchymal stromal cell conditioned media for lung disease: a systematic review and meta-analysis of preclinical studies

**DOI:** 10.1186/s12931-019-1212-x

**Published:** 2019-10-30

**Authors:** Chimobi Emukah, Evan Dittmar, Rija Naqvi, John Martinez, Alexis Corral, Axel Moreira, Alvaro Moreira

**Affiliations:** 1 Department of Pediatrics, Division of Neonatology, University of Texas Health-San Antonio, San Antonio, Texas 78229-3900 USA; 2 Department of Pediatrics, Texas Children’s Hospital, Houston, Texas USA

## Abstract

**Background:**

Inflammation plays an important role in the pathogenesis of many lung diseases. Preclinical studies suggest that mesenchymal stromal cell (MSC) conditioned media (CdM) can attenuate inflammation. Our aim was threefold: (1) summarize the existing animal literature evaluating CdM as a therapeutic agent for pediatric/adult lung disease, (2) quantify the effects of CdM on inflammation, and (3) compare inflammatory effects of CdM to MSCs.

**Methods:**

Adhering to the Systematic Review Protocol for Animal Intervention Studies, a systematic search of English articles was performed in five databases. Meta-analysis and meta-regression were performed to generate random effect size using standardized mean difference (SMD).

**Results:**

A total of 10 studies met inclusion. Lung diseases included bronchopulmonary dysplasia, asthma, pulmonary hypertension, and acute respiratory distress syndrome. CdM decreased inflammatory cells (1.02 SMD, 95% CI 0.86, 1.18) and cytokines (0.71 SMD, 95% CI 0.59, 0.84). The strongest effect for inflammatory cells was in bronchopulmonary dysplasia (3.74 SMD, 95% CI 3.13, 4.36) while pulmonary hypertension had the greatest reduction in inflammatory cytokine expression (1.44 SMD, 95% CI 1.18, 1.71). Overall, CdM and MSCs had similar anti-inflammatory effects.

**Conclusions:**

In this meta-analysis of animal models recapitulating lung disease, CdM improved inflammation and had an effect size comparable to MSCs. While these findings are encouraging, the risk of bias and heterogeneity limited the strength of our findings.

## Background

Lung disease is a major cause of morbidity and mortality [1, 2]. Respiratory conditions constitute 5 of the thirty most common causes of death worldwide [3]. As a result, the combined economic cost of respiratory disease in the UK in 2014 totaled £11.1 billion [4]. Thus, promoting healthy lives and minimizing lung infection/injury may preserve lifelong function and reduce chronic disease.

Inflammation plays a central role in the pathogenesis of many lung diseases [5–7]. An increasing number of studies have demonstrated a clear association between the degree of inflammation and lung structure/function [8–10]. Even an acute phase of inflammation has been shown to alter airway gas exchange, its ability to repair injured epithelium, and clear excess alveolar fluid [11, 12]. Consequently, utilizing therapies that target airway inflammation is a promising approach to mitigate airway disease.

The pleotropic effects of mesenchymal stem/stromal cells (MSCs) have positioned them as novel agents against lung disease [13–16]. Multiple studies highlight the anti-inflammatory and restorative tissue effects of MSCs [17–19]. Previously, it was believed that the advantages of stem cells lay in their engrafting ability; however, research now demonstrates their beneficial effects are via secretion of paracrine factors [20–22]. These bioactive factors can be collectively obtained and harbor the ability of molecular cueing to suppress inflammation, improve wound healing, angiogenesis, and stimulate regeneration [23]. To date, it is unknown which MSC factor(s) mediate their biologic action but collecting the milieu of secreted factors in their conditioned media (CdM) has proven successful in animal models of lung disease [24]. Advantages to the use of CdM over MSCs include biologic stability, cell-free transplantation, low risk of infection/recipient rejection, and ease for clinical scalability [25].

Currently, there has been no systematic review examining the therapeutic potential of MSC-derived CdM on inflammation in animal models recapitulating pediatric/adult lung disease. The results of this study are intended to identify the current research gaps and to generalize methods for CdM research. This systematic review and meta-analysis aims to: (i) methodically review the current literature describing the effects of CdM on animal models of pediatric/adult lung disease, (ii) quantify and analyze the effect of CdM on inflammation, (iii) compare inflammatory effects of CdM to MSCs, and (iv) identify limitations/research gaps that should be addressed in future preclinical studies.

## Methods

Our methods adhere to the guidelines established by the Systematic Review Centre for Laboratory Animal Experimentation (SYRCLE) and are described in Additional file 5: Table S1 [26]. Our protocol was registered through the Collaborative Approach to Meta-Analysis and Review of Data from Experimental Studies (CAMARADES) on August 7th, 2018.

### Literature search

A literature search was performed using MEDLINE’s database PubMed, Scopus, the Cumulative Index to Nursing and Allied Health Literature (CINAHL), Science Direct, and Google Scholar. Our major search criteria included “mesenchymal stem cell conditioned media,” “lung disease,” “animal,” and their synonyms (refer to Additional file 6: Table S2 for full criteria). Screenings by title/abstract and then by full-text review were conducted independently by three investigators (AM, CE, and ED). Reference lists from the included studies and relevant reviews were used in an effort to obtain additional sources for inclusion.

### Inclusion and exclusion criteria

In the current study, articles were included based on three main selection criteria: disease of interest, administration of MSC conditioned media, and assessment of inflammation. Specifically, in addressing disease, animal studies were included that focused on pediatric/adult models of bronchopulmonary dysplasia (BPD), asthma, pulmonary hypertension (PH), acute respiratory distress syndrome (ARDS), cystic fibrosis (CF), and pneumonia. Studies that addressed other pediatric or adult diseases were excluded. Of these, papers that addressed in vivo MSC-CdM administration were included, while papers solely addressing MSCs without CdM, or those using only ex vivo methods were excluded. Finally, articles were included only if their outcome assessment involved measures of inflammation. Papers with other outcome measures in addition to inflammation were still included.

### Primary and secondary endpoints

We defined our primary endpoint as inflammation, which is reported through inflammatory marker analysis after administration of conditioned media treatment. The markers were measured from any animal lung tissue and were grouped as either immune cells or inflammatory cytokines/proteins. Studies were excluded from the selection process if data pertaining to our primary outcome could not be obtained or if the study was not done on in-vivo or in animal models.

Our secondary outcome of lung architecture and/or function were not required for inclusion but was reported in many of the selected studies. The type of architectural and functional measures varies between articles but are used as additional measurements to gauge the efficacy of the conditioned media treatment.

This review presents the results of the primary endpoint analysis, while results from the secondary endpoint analysis will be reported in a future paper.

### Data extraction

Data was independently collected by two groups of investigators (ED and CE; AC and JM) and compared. A third investigator (AM) resolved any disagreements in the collected information. Extracted data included general study design (title, author, contact email, country, funding source(s), conflict(s) of interest), animal model characteristics (disease, disease induction, species, strain, age, gender, immune status), conditioned media characteristics (tissue source, dose, route of delivery, timing, frequency, CdM group name, disease group name), primary outcome measures (inflammatory markers and source, timing of outcome measurement), secondary outcome measures (lung architecture and/or lung function), other measures assessed, as well as a SYRCLE risk of bias assessment and MSC criteria assessment. Original data was gathered from graphs and plots using GetData graph digitizer version 2.26 when exact values were not accessible from the articles.

### Risk of bias

Risk of bias was assessed independently by two investigators (RN and AM) per the 2014 SYRCLE Risk of Bias tool for animal studies. The tool provides ten categories that evaluate bias related to selection, performance, detection, attrition and reporting. The responses of “yes” demonstrates a low risk of bias and “no” indicates a high risk. Studies that did not explicitly state its methods were marked as “unclear.”

### Data analysis

Meta-analysis was conducted using a random effects model to generate forest plots. The estimated effect size of CdM or MSC on immune cells or inflammatory cytokine was determined using standardized mean difference (SMD) and a 95% confidence interval (CI). Stratified effect was measured according to disease and by overall assessment of included lung disease.

Statistical heterogeneity between studies was calculated using the I^2^ metric. Potential sources of heterogeneity, if significant, were further investigated by meta-regression and subgroup analysis. Publication bias was examined using funnel plots with Egger’s tests.

All statistical analyses were performed using STATA v.13 (College Station, TX, USA).

## Results

### Study selection

**Fig. 1:**
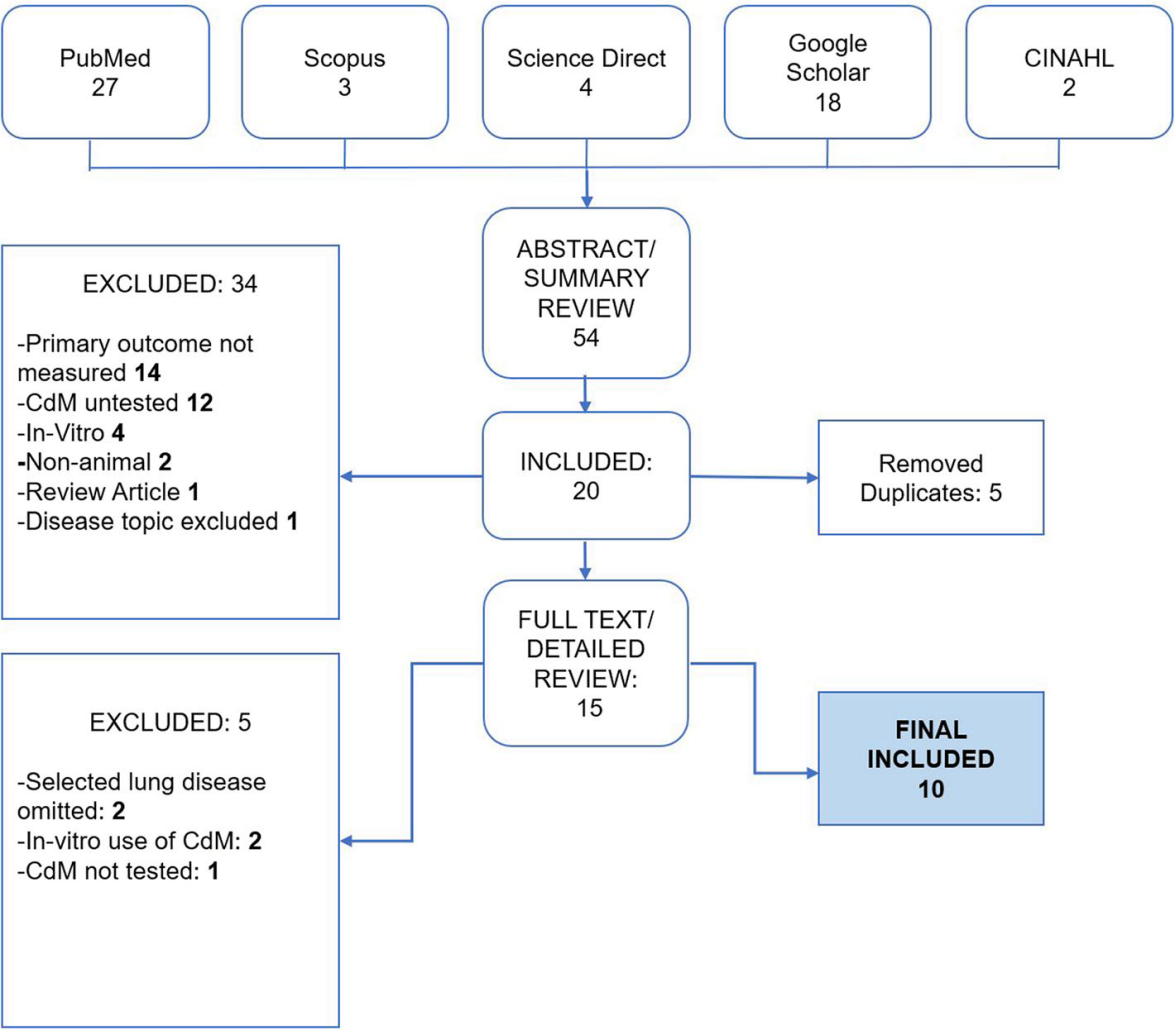
Flow diagram demonstrating study selection process

Our literature search produced 54 results from the utilized search terms. A total 49 results remained after removal of duplicate articles. After preliminary screening, 34 articles were excluded leaving 20 articles for full-text review. From the remaining, 10 published articles met defined criteria set and reported our primary outcome of inflammatory markers (Fig. 1 and Additional file 7: Table S3). All studies were reported in the review and meta-analysis.

#### Study characteristics

The studies included in this review were published between the years 2009 and 2017. Six of the included studies were from the United States, two were from Iran, one from Canada, and another from Japan. Relevant characteristics of these articles are described in Table 1. All of the studies used rodents as their animal model, with 60 % (*n* = 6) incorporating either C57/BL6 or FVB mice. Gender was reported in 90% (*n* = 9) of the studies, with the majority using only male rodents. Most of the studies used animals less than 12 weeks of age.

**Table 1:** Summary of study characteristics. Detailed summary of information extracted from included studies

Author (year)	Study Design		Animal Characteristics		Intervention Characteristics		Primary Outcome	Secondary Outcomes
	Disease	Disease	Animal	Age	Source;	Dose;	Inflammation	Lung
	Model	Induction	Model; Gender		(Origin)	Delivery; Timing; Frequency		Architecture/Function
Ionescu (2012)	ARDS	IT injection of 4 mg/kg LPS (*E. coli* 055:B5)	C57/BL6 mice; Male	Adult	Bone Marrow	30 μl; IT; 4 h post-LPS exposure; once	Cytokines	Lung sections
Lu (2015)	ARDS	Oropharyngeal aspiration 2 mg/kg LPS (E. Coli 0127:B8)	C57/BL6 mice; Male	Not Reported	Adipose Tissue	200 μl; IV tail vein; 4 h post-LPS exposure; once	TNF-alpha; IL-6; MIP-2; IL-10; VEGF	Alveolar wall sections
Wakaya-ma (2015)	ARDS	6 U/kg Bleomycin	C57/BL6J mice; Female	Adult	Human Exfoliated Deciduous Teeth	500 μl; IV jugular vein; 24 h post-bleomycin exposure; once	Cell surface markers; Cytokines	Fibrotic lung sections
Ahmadi (2016)	Asthma	IP administered OVA on days 1 and 8; day 14, exposure to 4% OVA nebulizer for 5 min for 18+/−1 days	Wistar rats; Male	Adult	Bone Marrow	50 μl; IV left femoral vein; 1 day post sensitization; once	CD3+; CD4+; CD8+	
Ahmadi (2017)	Asthma	IP administered OVA on days 1 and 8; day 14, 5 min exposure to 4% OVA nebulizer for 18+/−1 days	Wistar rats; Male	Adult	Bone Marrow	50 μl; IT; 1 day post sensitization; once	IL-4; IL-10	Emphysema;Atelectasis; Hyperemia; Epithelialization; Leukocyte Infiltration
Cruz (2015)	Asthma	5 μg Aspergillus fumigatus days 0 and 7 for sensitization; same inoculation on days 14 and 16 for challenge	C57/BL6 mice; Male	Adult	Bone Marrow	200 μl; IV tail vein; day 14 after aspergillus challenge; once	Neutrophils; Eosinophils; Macrophages; Lymphocytes; Cytokines	Lung sections; Airway resistance; Tissue resistance; lung elasticity
Aslam (2009)	BPD	Exposure to 75% O_2_ within 24 h of birth	FVB mice; male/female	Neonate	Bone Marrow	50 μl; IV superficial temporal vein; postnatal day 4; once	Macrophage; Neutrophils, Cytokines	Pulmonary arteriole; Lung alveoli; Medial wall thickness
Sutsko (2012)	BPD	2 weeks 90% O_2_ exposure	Sprague-Dawley rats; male/female	Neonate	Bone Marrow	50 μl; IT; postnatal day 9; once	IL-6/18 s; IL-1B/18 s; TTF-1/18 s; VEGF; angiopoietin-1	Alveolarization; lung vascular density
Lee (2012)	PH	8.5 ± 0.5% O_2_ exposure for 48 h	FVB mice; gender not reported	Adult	Bone Marrow	50 μl; IV left jugularvein; before 48 h hypoxia induction; once	MCP-1; FIZZ-1/HIMF	
Rathinas-abapathy (2016)	PH	50 mg/kg Monocrotaline	Sprague-Dawley rats; Male	Adult	Adipose Tissue	100 μl; IV jugular vein;14 days post-MCT exposure; once	Cell surface markers; Cytokines	Pulmonary vesselwall thickness; RV fibrosis

The pediatric/adult lung diseases that were represented in animal models included: asthma (*n* = 3), acute respiratory distress syndrome (*n* = 2), bronchopulmonary dysplasia (n = 2), and pulmonary hypertension (*n* = 2). Most studies used a single dose of 30 or 50 μl conditioned media, with an intravenous route being the most common delivery mode.

Inflammation was most commonly assessed via cytokine assays. A number of studies also investigated inflammation by flow cytometry. Eight of the articles examined secondary outcomes of interest.

### Conditioned media characteristics

Conditioned media criteria of the included studies are summarized in Additional file 8: Table S4. Allogeneic bone marrow tissue was the most common source of MSCs (70%). Six of the studies used Dulbecco’s Modified Eagle Medium for cell expansion and eight included antibiotic solution in their media.

### Risk of bias

Risk of bias was assessed via SYRCLE Risk of Bias tool for all ten studies included in our review. No study was judged as low risk across all ten domains. Only two stated that the allocation selection was random, thus many were evaluated as being “unclear” for sequence generation bias. The majority of experimental and control groups were reported to be similar at baseline, though two were deemed high risk for inconsistencies between groups. Ten percent (*n* = 1) of studies concealed allocation of treatment, whereas the rest did not explicitly state allocation method and were deemed “unclear”. None of the studies randomly housed the animals. Almost half (*n* = 4) of the studies randomly selected animals for outcome assessment, but only two studies reported blinding the outcome assessor. All studies were found to sufficiently report complete data and only one study was found to have reporting bias. Refer to Table 2.

**Table 2:** SYRCLE Risk of Bias Assessment for included studies

Author (Year)	Random sequence generation?	Groups similar at baseline?	Allocation concealed?	Animals randomly housed?	Blinding of caregivers and/or examiners?	Random selection for outcome assessment?	Blinding of outcome assessor?	Incomplete outcome data addressed?	Free from selective outcome reporting?	Free from other bias?
Ahmadi (2017)	Yes	Yes	Unclear	Unclear	Unclear	Yes	Unclear	Yes	Yes	Yes
Ahmadi (2016)	Yes	Yes	Unclear	Unclear	Unclear	Unclear	Unclear	Yes	Yes	Yes
Aslam (2009)	Unclear	No	Unclear	Unclear	Unclear	Unclear	Unclear	Yes	Yes	Yes
Cruz (2015)	Unclear	Yes	Unclear	Unclear	Unclear	Unclear	Unclear	Yes	No	Yes
Ionescu (2012)	Unclear	Yes	Unclear	Unclear	Unclear	Unclear	Unclear	Yes	Yes	Yes
Lee (2012)	Unclear	Unclear	Unclear	Unclear	Unclear	Unclear	Unclear	Yes	Yes	Yes
Lu (2015)	Unclear	Yes	Unclear	Unclear	Unclear	Unclear	Unclear	Yes	Yes	Yes
Rathinasabapathy (2016)	Unclear	Yes	Yes	Unclear	Yes	Yes	Yes	Yes	Yes	Yes
Sutsko (2012)	Unclear	No	Unclear	Unclear	Unclear	Yes	Yes	Yes	Yes	Yes
Wakayama (2015)	Unclear	Yes	Unclear	Unclear	Unclear	Yes	Unclear	Yes	Yes	Yes

### Stratified meta-analysis: Inflammatory outcomes

#### Immune Cells & CdM

**Fig. 2:**
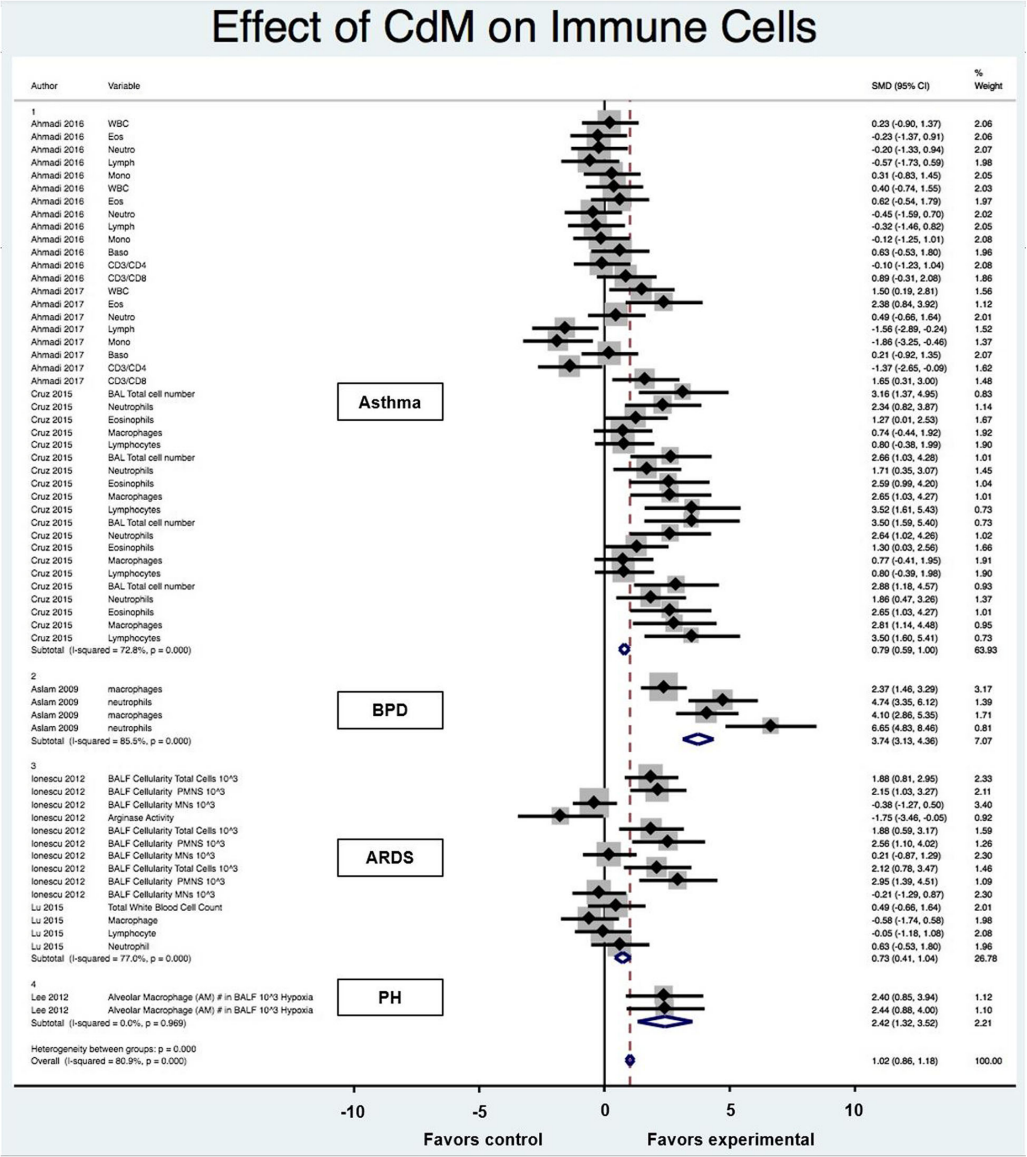
Effect size of CdM on immune cells stratified by disease process. Forest plots demonstrating SMD and 95% CI

Of the ten articles, seven evaluated the effect of CdM on immune cells. These studies incorporated the following pediatric/adult lung diseases: asthma, BPD, ARDS, and PH. An overall improvement in inflammatory cell recruitment occurred after CdM therapy with an SMD of 1.02 (95% CI 0.86, 1.18; 7 studies and 61 comparisons; Fig. 2). However, the heterogeneity between groups was significant (I^2^ = 80.9%; *p* < 0.001). When stratified and assessed by disease, conditioned media was favored over control for each disease but performed best for bronchopulmonary dysplasia (3.74 SMD; 95% CI 3.13, 4.36) followed by pulmonary hypertension (2.42 SMD; 95% CI 1.32,3.52). Administration of CdM in asthma animal models decreased eosinophilia but did not have much effect on lymphocytes. The total number of cells in the bronchoalveolar lavage of asthma and ARDS animals was reduced with CdM.

Stratification by animal model, study design, and intervention characteristics revealed significant differences in effect size for immune cells, seen in Table 3. Effect size was largest in BPD, studies conducted in mice (1.61 SMD; 95% CI 1.40, 1.82) and animals at neonatal age (3.74 SMD; 95% CI 3.13, 4.36). For intervention characteristics, effect size was larger in bone marrow derived conditioned media (1.10 SMD; 95% CI 0.93, 1.27). Also, effect size was larger when intervention was delivered intravenously (1.19 SMD; 95% CI 1.00, 1.39) and given greater than 72-h after injury (2.30 SMD; 95% CI 2.01, 2.59).

**Table 3:** Stratification of estimated CdM effect size on immune cells

	# Studies	# Comparisons	SMD (95% CI)	% Weight	I^2	p*	p**
Disease							
Asthma	3	41	0.79 (0.59, 0.10)	63.93	72.80	0.00	0.59
BPD	1	4	3.74 (3.13, 4.36)	7.07	85.50	0.00	
ARDS	2	14	0.72 (0.41, 1.04)	26.78	77.00	0.00	
PH	1	2	2.42 (1.32, 3.52)	2.21	0.00	0.97	
Animal model/species							
Mouse	5	40	1.61 (1.40, 1.82)	60.98	79.60	0.00	0.00
Rat	2	21	0.10 (-0.17, 0.36)	39.02	54.90	0.00	
Strain							
C57/BL6	4	36	1.33 (1.11, 1.56)	53.91	70.30	0.00	0.00
FVB	1	4	3.74 (3.13, 4.36)	7.07	85.50	0.00	
Wistar	2	21	0.10 (-0.17, 0.36)	39.02	54.90	0.00	
Gender							
Male	5	55	0.77 (0.60, 0.95)	90.71	73.50	0.00	0.01
Mixed	1	4	3.74 (3.13, 4.36)	7.07	85.50	0.00	
Not reported	1	2	2.42 (1.32, 3.52)	2.21	0.00	0.97	
Age							
Neonatal	1	4	3.74 (3.13, 4.36)	7.07	85.50	0.00	0.00
Adult	5	53	0.80 (0.70, 1.06)	84.90	74.50	0.00	
Not reported	1	4	0.12 (-0.46, 0.70)	8.03	0.00	0.45	
Intervention characteristics							
*Asthma*							0.18
Ovalbumin	2	21	0.10 (-0.17, 0.36)	39.02	54.90	0.00	
Aspergillus exposure	1	19	1.89 (1.56, 2.21)	24.91	41.60	0.03	
*ARDS*							
LPS	2	14	0.73 (.41, 1.04)	26.78	77.00	0.00	
*BPD*							
75% hyperoxia	1	4	3.74 (3.13, 4.36)	7.07	85.50	0.00	
*PH*							
Hypoxia	1	2	2.42 (1.32, 3.52)	2.21	0.00	0.97	
Source							
Bone marrow	6	57	1.10 (0.93, 1.27)	91.97	81.40	0.00	
Adipose tissue	1	4	0.12 (-0.46, 0.70)	8.03	0.00	0.45	0.14
Origin							
Allogenic	7	61	1.02 (0.86, 1.18)	100.00	80.90	0.00	0.00
Dose							
200ul	2	24	1.46 (1.17, 1.74)	32.95	63.20	0.00	0.00
50ul	4	27	0.74 (0.50, 0.97)	48.30	86.30	0.00	
30ul	1	10	0.99 (1.17, 1.74)	18.75	81.20	0.00	
Delivery							
Intravenous	5	43	1.19 (1.00, 1.39)	68.50	80.20	0.00	0.08
Intratracheal	2	18	0.64 (0.35, 0.93)	31.50	81.70	0.00	
Timing of treatment							
<72hr	5	37	0.42 (0.22, 0.62)	68.01	70.80	0.00	0.00
>72hr	2	24	2.30 (2.01, 2.59)	31.99	71.40	0.00	

*p refers to *p* value within subgroups

**p refers to *p* value between subgroups

#### Cytokines & CdM

**Fig. 3:**
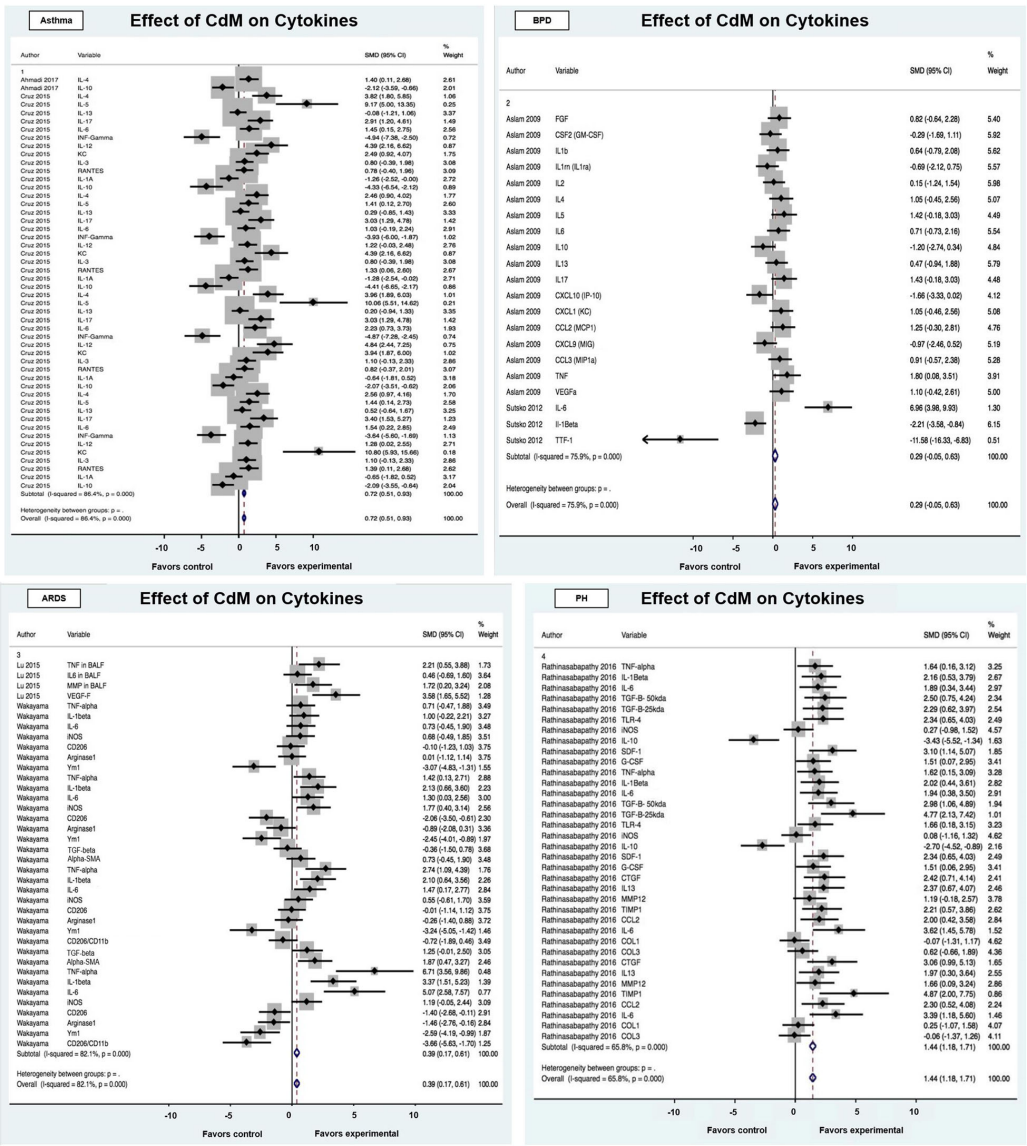
Effect size of CdM on inflammatory cytokines stratified by disease process. Forest plots demonstrating SMD and 95% CI

Of the ten articles, seven evaluated the effect of CdM on inflammatory cytokines. The studies included animal models mimicking asthma, BPD, ARDS, and PH. Like the outcomes in immune cells, CdM had an overall positive effect on cytokine analysis (SMD of 0.71 95% CI, 0.59, 0.84; Fig. 3). Significant heterogeneity (I^2^ = 81.9%; *p* < 0.001) was observed. When assessed by disease, conditioned media decreased cytokine expression for each disease except for bronchopulmonary dysplasia. Among the cytokines, CdM lessened the expression of tumor necrosis factor alpha, interleukin beta, transforming growth factor beta, and increased anti-inflammatory factors interleukin 10. In asthma preclinical studies, the treatment effect shifted away from a T cell helper 2 biased inflammation (e.g. interleukin 5, 4, and interferon gamma) [27]. Stratification by animal model, study design, and intervention characteristics were also performed for the assessment of cytokines, seen in Table 4. Effect size was largest for pulmonary hypertension (1.44 SMD; 95% CI 1.18, 1.71). The effect size was highest in mice (1.22 SMD; 95% CI 0.97, 1.47) and male animals (1.02 SMD; 95% CI 0.86, 1.18). For intervention characteristics, effect size was larger in LPS induced injury models (1.56 SMD; 95% CI 0.82, 2.31). Adipose tissue derived conditioned media (1.46 SMD; 95% CI 1.21, 1.71) had larger effect sizes at a dose of 100μL. Similar to the immune cell assessment, intravenously delivered intervention (0.73 SMD; 95% CI 0.60, 0.85), and delivery greater than 72-h after injury (0.92 SMD; 95% CI 0.77, 1.07) had the largest effect size.

**Table 4:** Stratification of estimated CdM effect size on inflammatory cytokines

Disease							
Asthma	2	50	0.72 (0.51, 0.93)	34.70	86.40	0	0.29
BPD	2	21	0.29 (−0.05, 0.63)	13.00	75.90	0	
ARDS	2	38	0.39 (0.17, 0.61)	31.19	82.10	0	
PH	1	36	1.44 (1.18, 1.71)	21.11	65.80	0	
Animal model/species							
Mouse	4	104	1.22 (0.97, 1.47)	23.75	82.40	0	0.13
Rat	3	41	0.56 (0.42, 0.70)	76.25	79.10	0	
Strain							
C57/BL6	3	86	0.58 (0.43, 0.73)	64.29	84.80	0	0.29
FVB	2	18	0.43 (0.07, 0.78)	11.97	36.00	0.07	
Sprague-Dawley	1	39	1.32 (1.06, 1.58)	22.14	77.70	0	
Wistar	1	2	(−)0.14 (−1.10, 0.83)	1.60	92.00	0	
Gender							
Male	4	90	1.02 (0.86, 1.18)	58.53	81.90	0	0.03
Mixed	2	21	0.29 (−0.05, 0.63)	13.00	75.90	0	
Female	1	34	0.28 (0.05, 0.51)	28.47	82.50	0	
Age							
Neonatal	1	21	0.29 (−0.05, 0.63)	13.00	75.90	0	0.13
Adult	6	120	0.75 (0.62, 0.89)	84.27	92.00	0	
NR	1	4	1.56 (0.82, 2.31)	2.73	64.20	0.04	
Intervention characteristics							
*Asthma*							0.26
Ovalbumin	1	2	(−)0.14 (−1.10, 0.83)	1.60	92.00	0	
Aspergillus exposure	1	48	0.76 (0.55, 0.98)	33.09	86.40	0	
*ARDS*							
LPS	1	4	1.56 (0.82, 2.31)	2.73	64.20	0.04	
Bleomycin	1	34	0.28 (0.05, 0.51)	28.47	82.50	0	
*BPD*							
75% hyperoxia	1	18	0.43 (0.07, 0.78)	11.97	36.00	0.07	
90% hyperoxia	1	3	(−)1.31(−2.51, −0.10)	1.03	95.70	0	
*PH*							
Moncrotaline/bleomycin	1	36	1.44 (1.18, 1.71)	21.11	65.80	0	
Source							
Bone marrow	4	71	0.60 (0.43, 0.78)	47.70	84.40	0	0.42
Adipose tissue	2	40	1.46 (1.21, 1.71)	23.83	64.80	0	
Decidious teeth	1	34	0.28 (0.05, 0.51)	28.47	82.50	0	
Origin							
Allogenic	6	111	0.89 (0.74, 1.03)	71.53	81.30	0	0.11
Xenogenic	1	34	0.28 (0.05, 0.51)	28.47	82.50	0	
Dose							
500ul	2	34	0.28 (0.05, 0.51)	28.47	82.50	0	0.63
200ul	1	52	0.82 (0.62, 1.03)	35.82	85.80	0	
100ul	1	36	1.44 (1.18, 1.71)	21.11	65.80	0	
50ul	3	23	0.24 (−0.08, 0.56)	14.61	77.20	0	
Delivery							
Intravenous	6	143	0.73 (0.60, 0.85)	98.40	81.80	0	0.45
Intratracheal	1	2	(−)0.14 (−1.10, 0.83)	1.60	92.00	0	
Timing							
<72 h	3	43	0.31 (0.10, 0.52)	33.83	84.90	0	0.08
>72 h	4	102	0.92 (0.77, 1.07)	66.17	79.80	0	

#### Immune Cells & MSCs

**Fig. 4:**
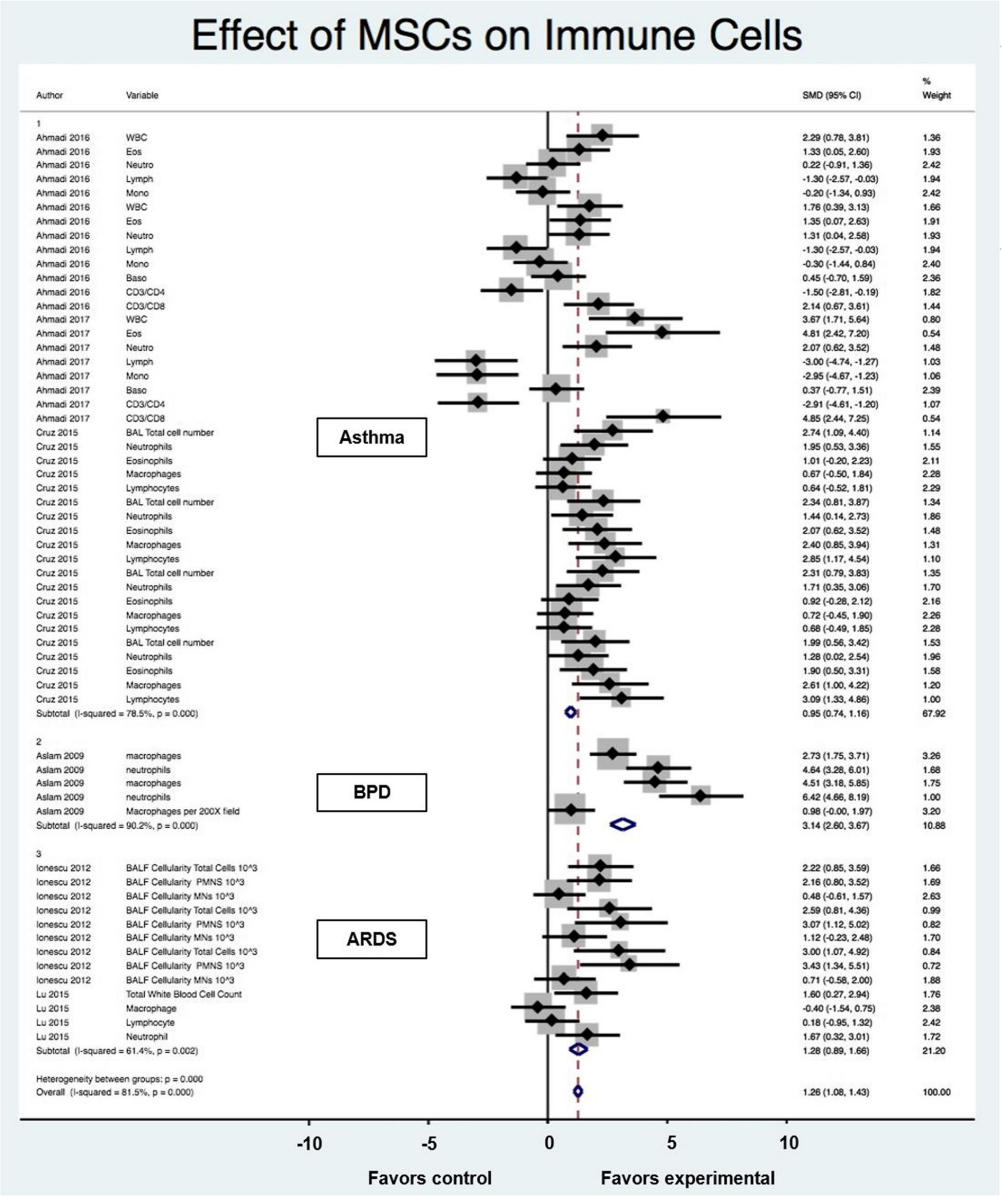
Effect size of MSCs on immune cells stratified by disease process. Forest plots demonstrating SMD and 95% CI

From the ten articles, six evaluated the effects of MSC on immune cells (Fig. 4). The studies highlighted lung disease in animal models of asthma, bronchopulmonary dysplasia, and acute respiratory distress syndrome. MSCs had an overall positive effect and was favored over the control with a SMD of 1.26 (95% CI 1.08, 1.43). However, the heterogeneity between groups was significant (I^2^ = 81.5%; *p* < 0.001). When assessed for each disease, the MSC treatment was favored over control for each model but performed best for bronchopulmonary dysplasia (3.14 SMD; 95% CI 2.60, 3.67). Administration of MSCs in asthma animal models decreased eosinophilia, neutrophilia and overall WBC. The total number of cells in the bronchoalveolar lavage was decreased in all studies.

#### Cytokines & MSCs

**Fig. 5:**
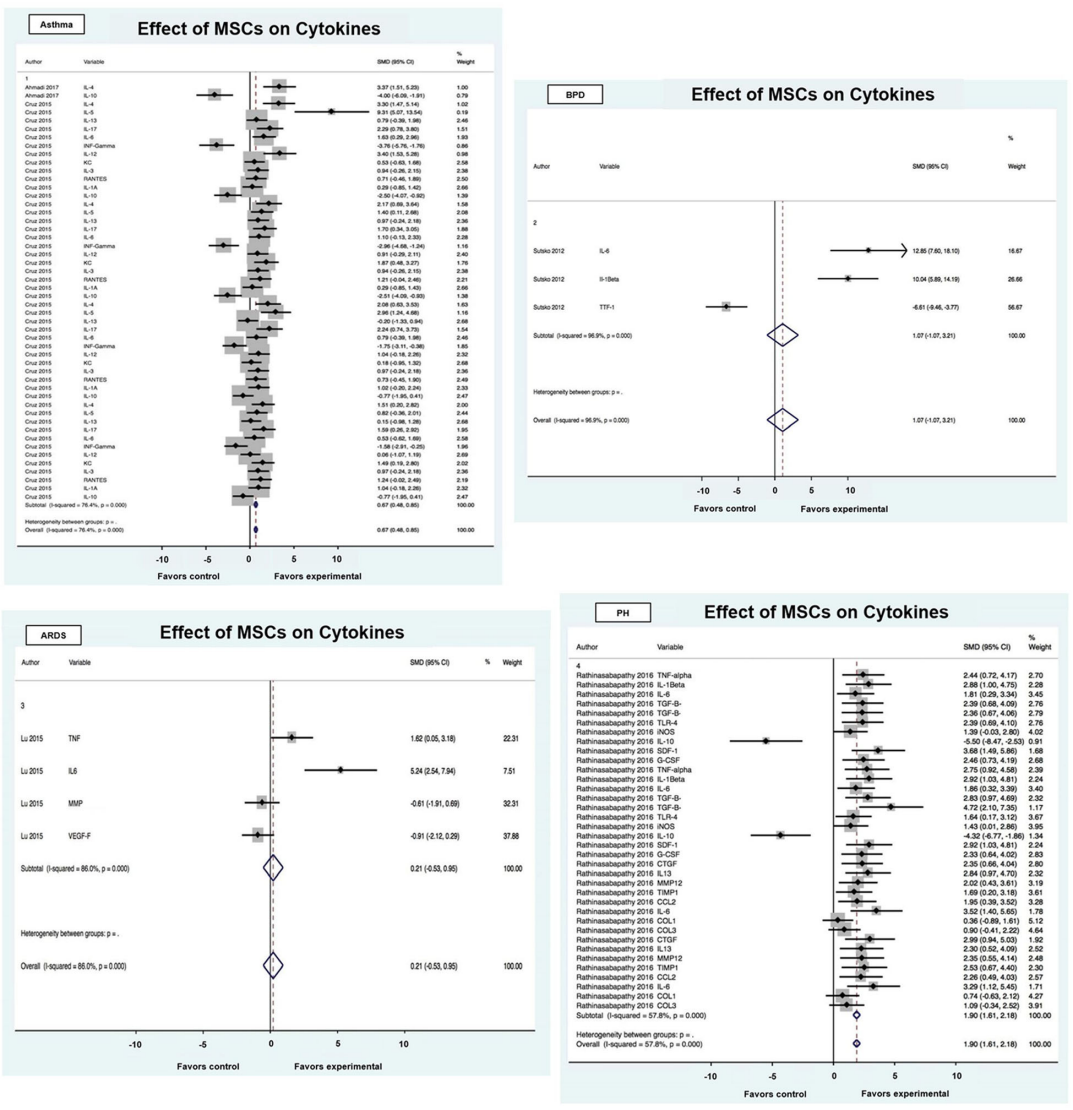
Effect size of MSCs on immune cells stratified by disease process. Forest plots demonstrating SMD and 95% CI

Five of the ten articles assessed the effect of MSCs on inflammatory cytokines and included animal models of the following lung diseases: asthma, BPD, ARDS, and PH (Fig. 5). The effect of the MSCs on the injury model evaluated via cytokine analysis was positive overall with an SMD of 1.00 (95% CI 0.85, 1.15). Significant heterogeneity (I^2^ = 78.7%; *p* < 0.001) was observed. The intervention was most effective for pulmonary hypertension (1.44 SMD; 95% CI 1.18, 1.71). Among the cytokines, MSCs reduced the expression of interleukin 6, interferon gamma, and increased interleukin 10. In bronchopulmonary dysplasia, MSC treatment improved the expression of thyroid transcription factor 1, a gene important in normal lung embryogenesis and surfactant production [28]. Like CdM effects in asthma, MSCs shifted away from a T cell helper 2 biased inflammation.

### CdM vs MSC comparison

#### Immune cells: CdM v. MSCs

In assessing the overall effects on immune cells in asthma, BPD, and ARDS, the effectiveness of CdM vs MSC treatment was comparable [SMD of − 0.15 (95% CI -0.31, 0.01)]. Graphical representation available in Additional file 1: Figure S1. Asthma slightly favored CdM but BPD and ARDS were slightly improved after MSC treatment.

#### Cytokines: CdM v. MSCs

In evaluating treatment effect on cytokines, overall assessment showed similar results between CdM vs MSC [SMD of − 0.10 (95% CI -0.24, 0.04)]. Again, asthma favored CdM but MSC therapy was better for BPD and PH (Additional file 2: Figure S2).

#### Subgroup analysis in ARDS

ARDS was the only disease that had differing tissue sources of CdM (bone marrow vs. adipose vs. decidual teeth) with more than one comparison study for immune cells and inflammatory cytokines. Additional file 3: Figure S3 shows that bone marrow CdM had a greater reduction in inflammatory cells when compared to adipose-derived CdM (SMD of 0.99 vs. SMD of 0.12) In contrast, adipose-generated CdM was superior to decidual teeth CdM in respects to inflammatory cytokines (SMD of 1.56 vs SMD of 0.28, Additional file 4: Figure S4).

### Narrative findings

CdM use in assessing emphysema, atelectasis, hyperemia, epithelialization, and leukocyte infiltration were observed by one study. The results were not included in the statistical analysis due to the nature of reported outcomes. However, Ahmadi et al. found a significant decrease in pathological changes in CdM treated but noted that MSCs produced a more efficient amelioration of these pathological changes [29]. Sutsko et al and Aslam et al reported improvement in histologic measures of alveolarization [30, 31]. Additionally, Aslam et al documented decreased muscularization of intrapulmonary arterioles after hyperoxic injury. Studies completed by Ionescu and Wakayama similarly suggest improvements in lung fibrosis and septal thickening [32, 33]. These histological findings support the idea of conditioned media’s pulmonary restorative nature.

### Meta-regression analysis

Meta-regression was performed to simultaneously survey the impact of all variables on study effect. For immune cell assessment, animal species and strain, gender, age at lung injury induction, MSC origin, and timing of intervention (6 of 11 themes) were significant sources of heterogeneity (*p* < 0.05) (Table 3). As for the cytokine assay assessment, gender was the only significant source for heterogeneity (*p* < 0.05) (Table 4).

### Publication bias

**Fig. 6:**
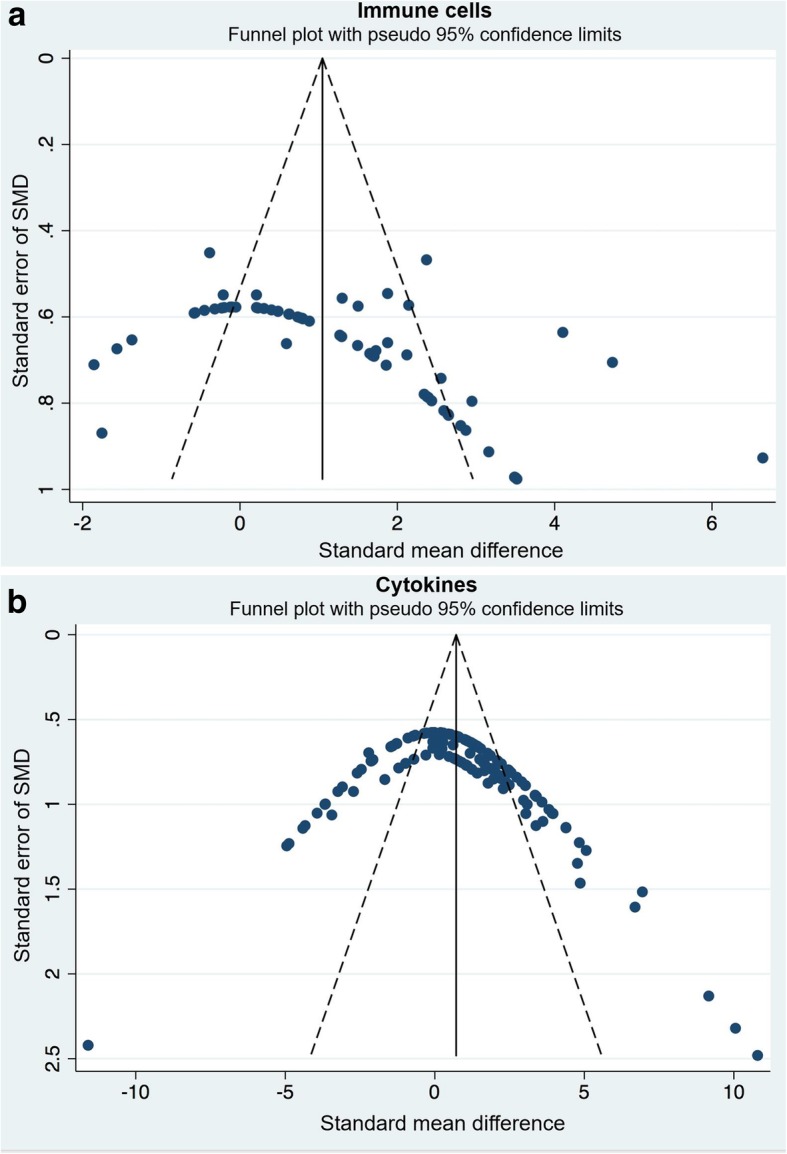
Funnel plots demonstrating publication bias from included studies. Funnel plots for (**a**) immune cells, and (**b**) inflammatory cytokines

Funnel plots were created to examine the effect of study qualities and heterogeneity on publication bias (Fig. 6a & b). Asymmetry was detected in funnel plots of immune cells and cytokines, indicating the presence of publication bias in these studies. Egger’s tests were performed to formally detect statistical asymmetry, with a null hypothesis denying the existence of small study effects. The *p* value was < 0.05 for all tests, indicating strong evidence to reject the null hypothesis in favor of the alternative (i.e. small study effect does exist).

## Discussion

The goal of systematic reviews and meta-analyses of preclinical studies is to provide a cumulative evaluation of treatments across multiple studies and to identify areas for improvement in research [26, 34, 35]. These efforts are intended to guide future studies and increase the translatability into the clinical setting. In preclinical settings, MSCs improve survival, pulmonary function, and protect against structural changes via secretion of paracrine factors. Thus, in theory, MSC-derived CdM may have similar reparative, regenerative, and restorative properties as the cells themselves. However, to date there has been no systematic analysis of CdM as a potential therapeutic option for inflammation in animal studies modeling lung disease. This is the first preclinical meta-analysis indicating that CdM has therapeutic potential in not only attenuating inflammation in animal models of pediatric/adult lung disease but are oftentimes as effective as MSCs.

In line with our CdM effects on immune cells, Augustine and colleagues also demonstrated a decrease in alveolar macrophage number after treatment of BPD animals with MSCs (1.90 SMD for MSCs vs. 2.42 SMD for CdM) [36]. The mixed results on neutrophil and white cell counts were also observed in this meta-analysis. Similarly, a downregulation of many of the cytokines seen in our review was observed by Augustine’s group. For example, IL-6 in their study had an SMD of 2.28, while ours had an SMD of 2.23, favoring treatment with regenerative cells. A decrease in the pro-inflammatory cytokine IL-6 was also appreciated in a study examining CdM as a therapy for renal fibrosis and in a pulmonary fibrosis meta-analysis conducted by Srour et al [37]*.* Our overall findings that CdM attenuates inflammation was also corroborated by a review article by Mohammadipoor et al wherein they describe the anti-inflammatory effects of CdM in pulmonary disease [38].

Other inflammatory cytokines that were decreased in our review included IL-1β and TNFα. A study by Platas et al found a decrease in the expression of IL-1β from osteoarthritic cells after exposure to stromal cell conditioned media [39]. A similar reduction in IL-1β (3.17 SMD for MSCs vs. 3.37 SMD for CdM) and TNFα was mirrored in the preclinical BPD meta-analysis by Augustine et al. TNFα inhibition was also seen in a study performed in an animal model of arthritis receiving CdM [40].

Herein we conduct the first preclinical meta-analysis comparing CdM to MSC as therapies for lung disease. Finding that CdM was as effective as MSCs, in an analysis of 10 studies, is a noteworthy finding in the field. Even more impressive is the fact that both therapies had the largest effect sizes on immune cells and cytokines for the same diseases (BPD and PH, respectively). Our results open opportunities for the development of therapies that may not require the cells. Clinically speaking, this suggests the potential preparation of a cell-free “drug” that can be produced, tested, and stored prior to use.

Our meta-analysis shows the need for further study to determine the ideal dosage and type of MSC-derived CdM. While a dosage of 200 μl were favored in the immune response, 100 μl dosages were favored in evaluation of cytokines. Similarly, there were discrepancies in the most effective tissue source of CdM. In immune cell evaluation, bone marrow had the best outcome whereas adipose performed better for cytokine evaluation. These mixed results need to be further investigated in future studies to determine optimal CdM treatment for lung disease. Consequently, our meta-analysis should be used to guide future preclinical studies in efforts to move these findings into a clinical setting.

### Clinical relevance

As of September 2nd, 2019, a search of the four lung diseases and stem cells in clinicaltrials.gov yielded 33 studies (Additional file 9: Table S5). While a similar search was undertaken with CdM, no results were retrieved. More than half of the cell-based studies registered were targeting BPD followed by eight intervention studies in ARDS. Aside from BPD, the population of interest in the clinical trials includes adults.

Source of the stromal cells incorporated adipose, bone, umbilical cord, and menstrual tissue. The majority of studies (88%) will use/used allogeneic stem cell transplants with a dose ranging from 500 k cells/kg of body weight to a total of 160 million cells. Of the eight completed studies, six described their early phase findings.

1. ARDS-no treatment-related adverse events and cells may improve markers of systemic injury/inflammation
2. PH-no severe adverse events in one study, while the other study had a death of one at discharge that the safety monitoring board stated may have possibly been related to cell therapy; both studies with improvement in walking distance
3. BPD-no serious adverse events in studies with some preliminary results pointing towards a reduction in some markers of lung inflammation, one trial did not show a difference in the development of severe BPD, yet one trial suggested a decrease in home oxygen use and improved developmental parameters

Yet some of these clinical findings appear promising, we must bear in mind that these are early phase studies and conducted in a small number of patients. Although none of the studies incorporated the use of CdM one study in particular (NCT03857841) plans to utilize bone marrow-derived MSC extracellular vesicles. These secreted particles are commonly found in CdM and studies as such may pave the road to future clinical trials utilizing regenerative cell-free therapies. Potential advantages in using CdM over MSCs are summarized in Additional file 10: Table S6. After further investigation, we did find one study that is examining the therapeutic potential of umbilical cord blood-derived stem cell conditioned media for alopecia (NCT03676400).

### Translational implications

In spite of positive effects of regenerative cells for lung disease in animal studies many foundational questions remain. Our systematic review was hindered by the small number of studies and the large heterogeneity regarding CdM source, dose, and animal models of lung disease. Unfortunately, our review was not able to provide clear directions or recommendations for future clinical trials. However, generalizations can be extrapolated and include the following: i) CdM and MSCs improve inflammation in rodent models of pediatric/adultlung disease, ii) CdM and MSCs may be equally efficacious, iii) the intravenous route was superior in reducing inflammation compared to the intratracheal route, iv) administration of CdM after 72 h of the initial pulmonary insult may be preferred, and v) the source, volume, concentration, and frequency of CdM therapy is unclear.

Future translational studies should reduce bias and attempt to standardize many of the characteristics (source, dose, frequency, measures, etc) that may impact the efficacy of regenerative cell-based/derived products. A novel approach undertaking some of these knowledge gaps can be observed in cardiovascular research. The Transnational AllianCe for regenerative Therapies In Cardiovascular Syndromes (TACTICS) group has called for a paradigm shift in preclinical research to facilitate successful translation into the clinics. They recommend scientific collaborative efforts that includes sharing of protocols, knowledge, and multicenter animal studies complemented with confirmatory studies in large animals [41].

There are several limitations to our systematic review and meta-analysis. First, we had a limited number of studies and experiments. Second, we incorporated multiple animal models of pediatric/adult lung disease which most likely contributed to the high heterogeneity. Our interest was to examine the effects of regenerative therapies in animal models of lung disease; however, many of the studies used were exclusively performed in adult animals. Thus, our review did not include the wide spectrum of respiratory disease (e.g., chronic obstructive pulmonary diseases, influenza, tuberculosis, and lung cancer). This discrepancy may impede the translation of our findings. Third, none of the studies had a low risk of bias. Next, implications from this work must be viewed with caution given that the nature of the studies were all completed in small animal models of pediatric/adult lung disease. The final major barrier to our study was the current lack of systemic review or meta-analysis using CdM on models of lung disease. While the field is saturated with information on the use of MSC on lung disease, there was much less evidence on the effects of CdM, which inhibited our ability to corroborate/refute our findings.

## Conclusion

In sum, this systematic review and meta-analysis is the first to summarize and quantify the effects of CdM as a therapeutic agent to ameliorate lung and systemic inflammation. CdM improved inflammation and was as effective as MSCs. While these findings are encouraging, we must acknowledge that the studies were performed in small animals and oftentimes positive results in rodents do not translate to the patient bedside. Studies included in this review had high heterogeneity and an overall unclear risk of bias. Further analysis must be done to move the field forward, such as determining the ideal site of CdM delivery, dosage, and timing of treatment according to disease.

## Supplementary information

**Additional file 1: Figure S1.** Effect size of CdM vs. MSC on immune cells stratified by disease process. Forest plots demonstrating SMD and 95% CI.

**Additional file 2: Figure S2.** Effect size of CdM vs. MSC on inflammatory cytokines stratified by disease process. Forest plots demonstrating SMD and 95% CI.

**Additional file 3: Figure S3.** Effect size of CdM tissue source on immune cells in ARDS. Forest plots demonstrating SMD and 95% CI.

**Additional file 4: Figure S4.** Effect size of CdM tissue source on inflammatory cytokines in ARDS. Forest plots demonstrating SMD and 95% CI.

**Additional file 5: Table S1.** SYRCLE 2014 protocol format.

**Additional file 6: Table S2.** Literature search terms.

**Additional file 7: Table S3.** Number of animals in studies.

**Additional file 8: Table S4.** CdM characteristics.

**Additional file 9: Table S5.** Current clinical trials in asthma, BPD, ARDS, PH.

**Additional file 10: Table S6.** Advantages of CdM vs. MSCs.

## Data Availability

The datasets generated and/or analyzed during the current study are available upon reasonable request.

## Abbreviations

ARDS: Acute Respiratory Distress Syndrome
ARDS: Acute respiratory distress syndrome
BPD: Bronchopulmonary Dysplasia
BPD: Bronchopulmonary dysplasia
CAMARADES: Collaborative Approach to Meta-Analysis and Review of Data from Experimental Studies
CdM: Conditioned media
CF: Cystic fibrosis
CI: Confidence interval
CINAHL: Cumulative Index to Nursing and Allied Health Literature
IL-1β: Interleukin-1 beta
IP: Intraperitoneal
IT: Intratracheal
IV: Intravenous
LPS: Lipopolysaccharide
MCT: Monocrotaline
MSCs: Mesenchymal stem/stromal cells
OVA: Ovalbumin
PH: Pulmonary Hypertension
PH: Pulmonary hypertension
PN: Postnatal
RV: Right Ventricle
SMD: Standardized mean difference
SYRCLE: Systematic Review Centre for Laboratory Animal Experimentation
TNFα: Tumor necrosis factor alpha
