# Supplementary figures and images for: Mesenchymal stromal cell conditioned media for lung disease: a systematic review and meta-analysis of preclinical studies
Source: Respir Res. 2019 Oct 30;20:239. doi: 10.1186/s12931-019-1212-x (PMC6822429; doi:10.1186/s12931-019-1212-x)

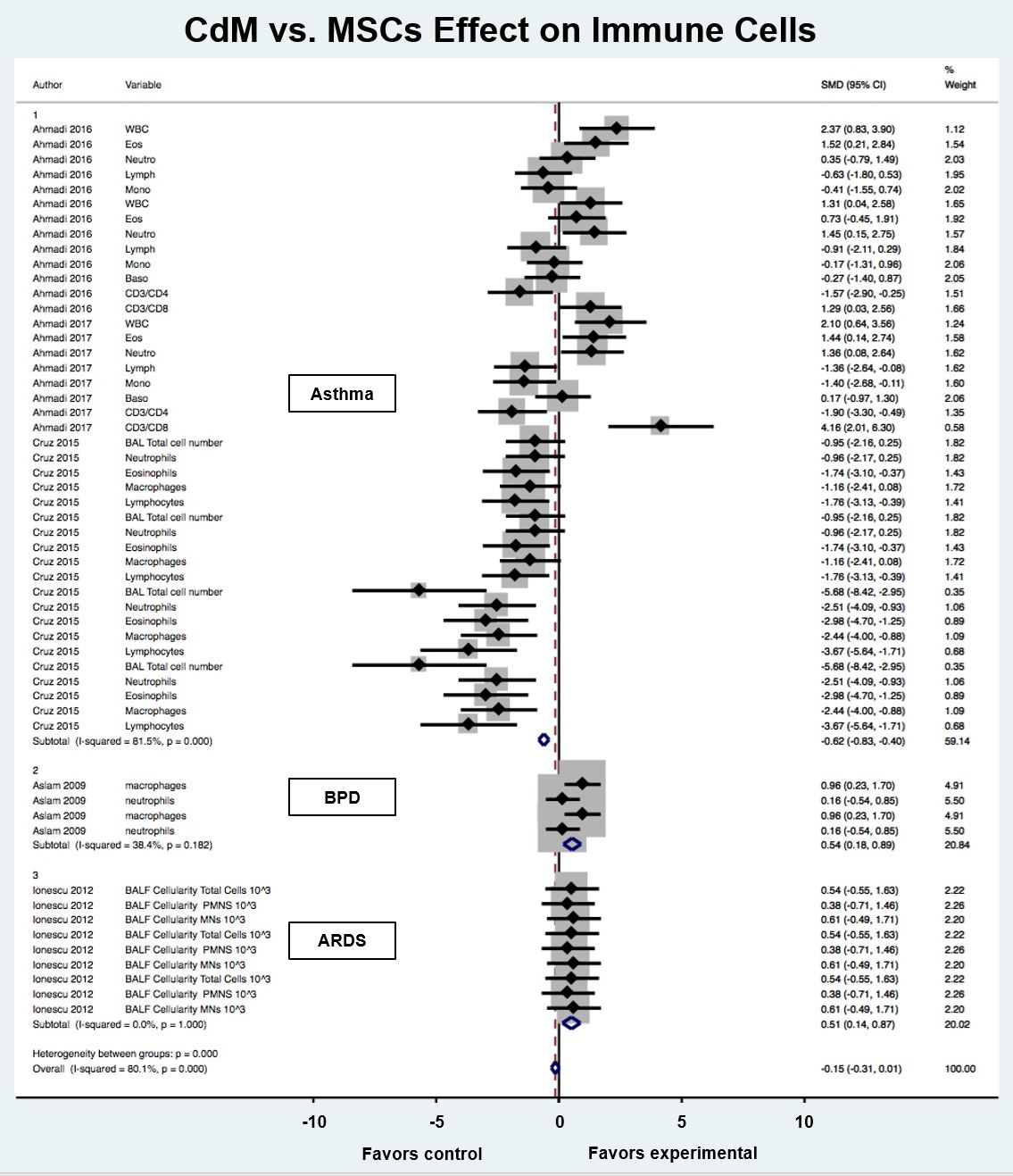

Supplement: Supplementary file 1 — Additional file 1: Figure S1. Effect size of CdM vs. MSC on immune cells stratified by disease process. Forest plots demonstrating SMD and 95% CI. [file 12931_2019_1212_MOESM1_ESM.jpg]

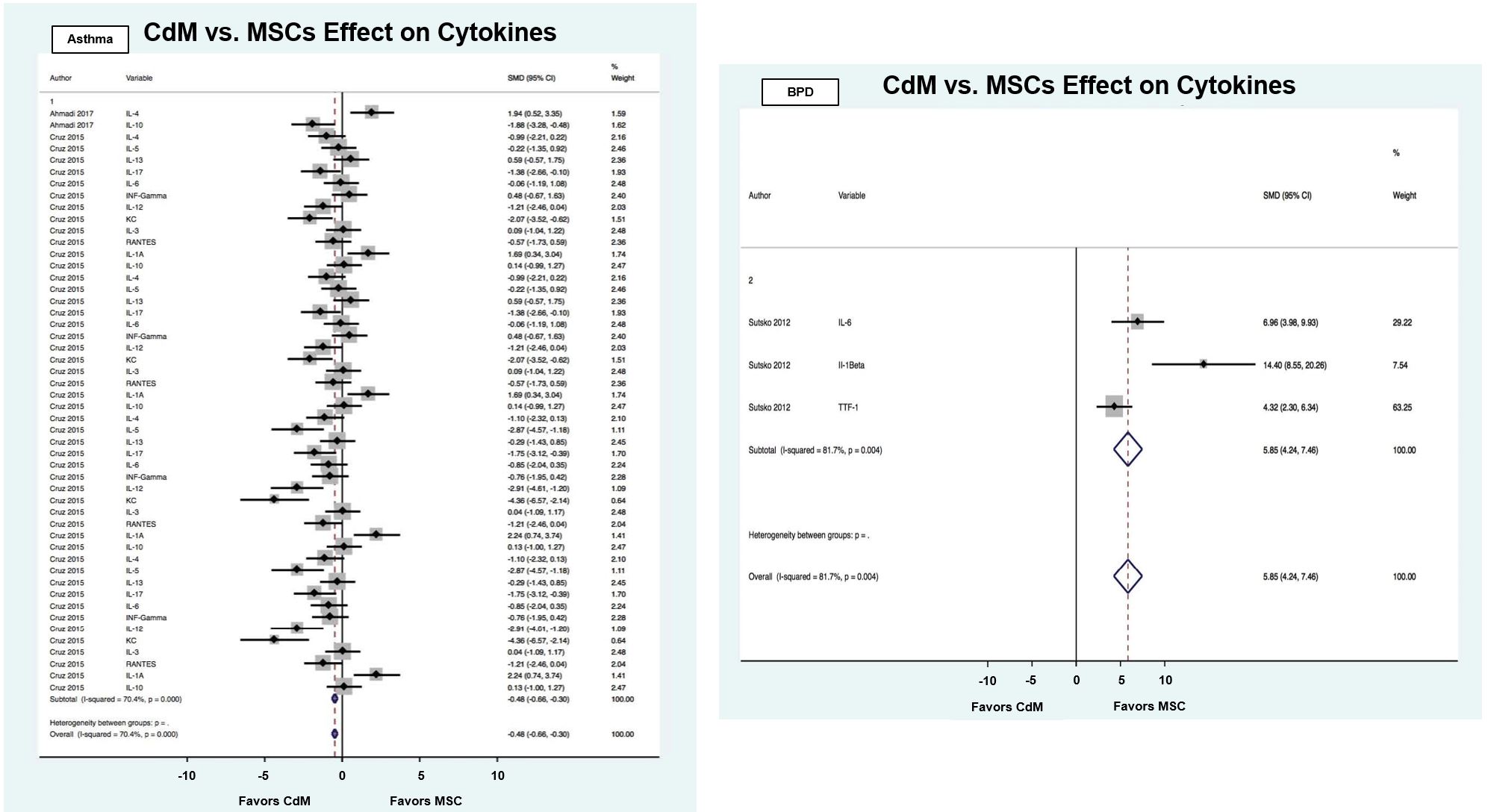

Supplement: Supplementary file 2 — Additional file 2: Figure S2. Effect size of CdM vs. MSC on inflammatory cytokines stratified by disease process. Forest plots demonstrating SMD and 95% CI. [file 12931_2019_1212_MOESM2_ESM.jpg]

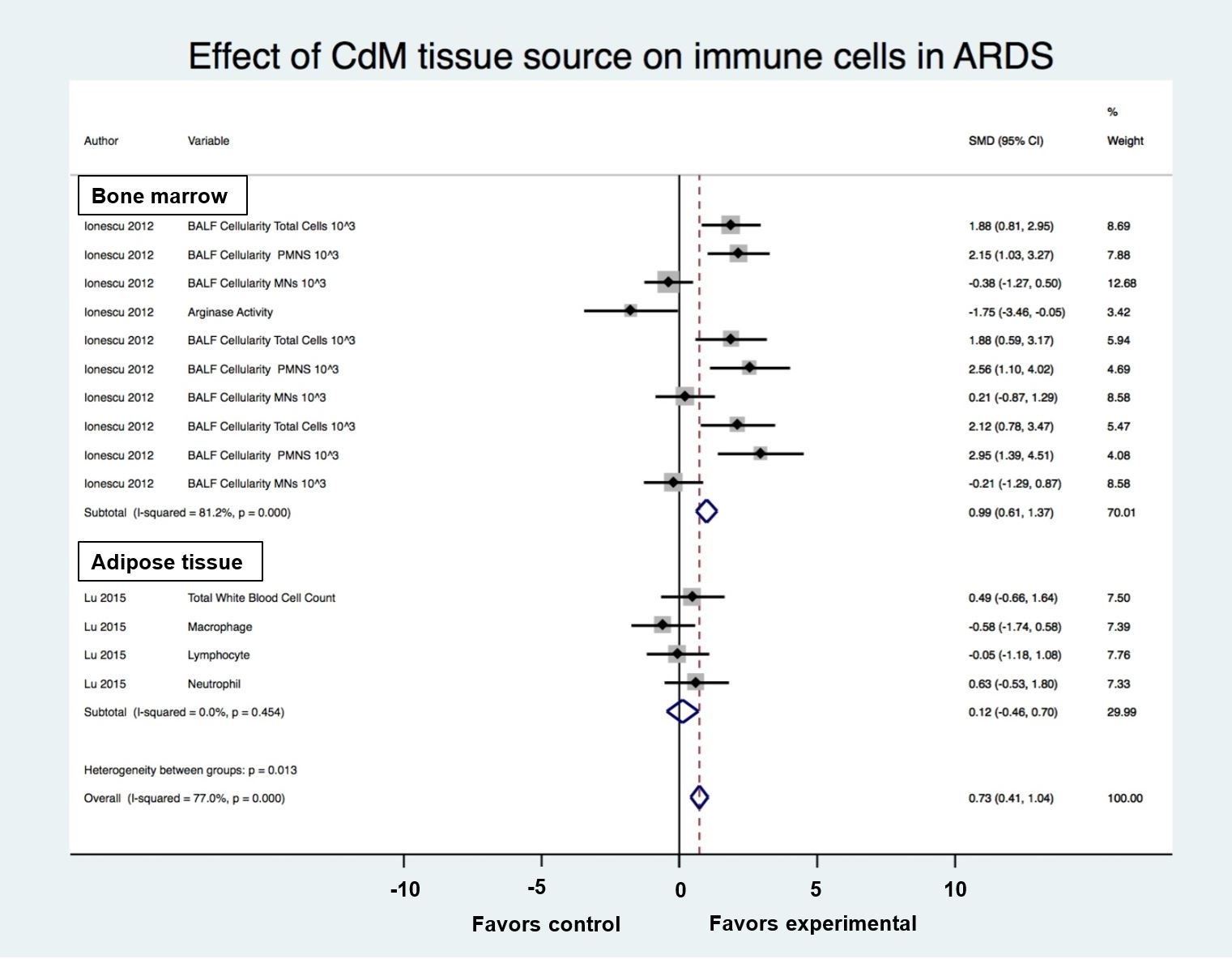

Supplement: Supplementary file 3 — Additional file 3: Figure S3. Effect size of CdM tissue source on immune cells in ARDS. Forest plots demonstrating SMD and 95% CI. [file 12931_2019_1212_MOESM3_ESM.jpg]

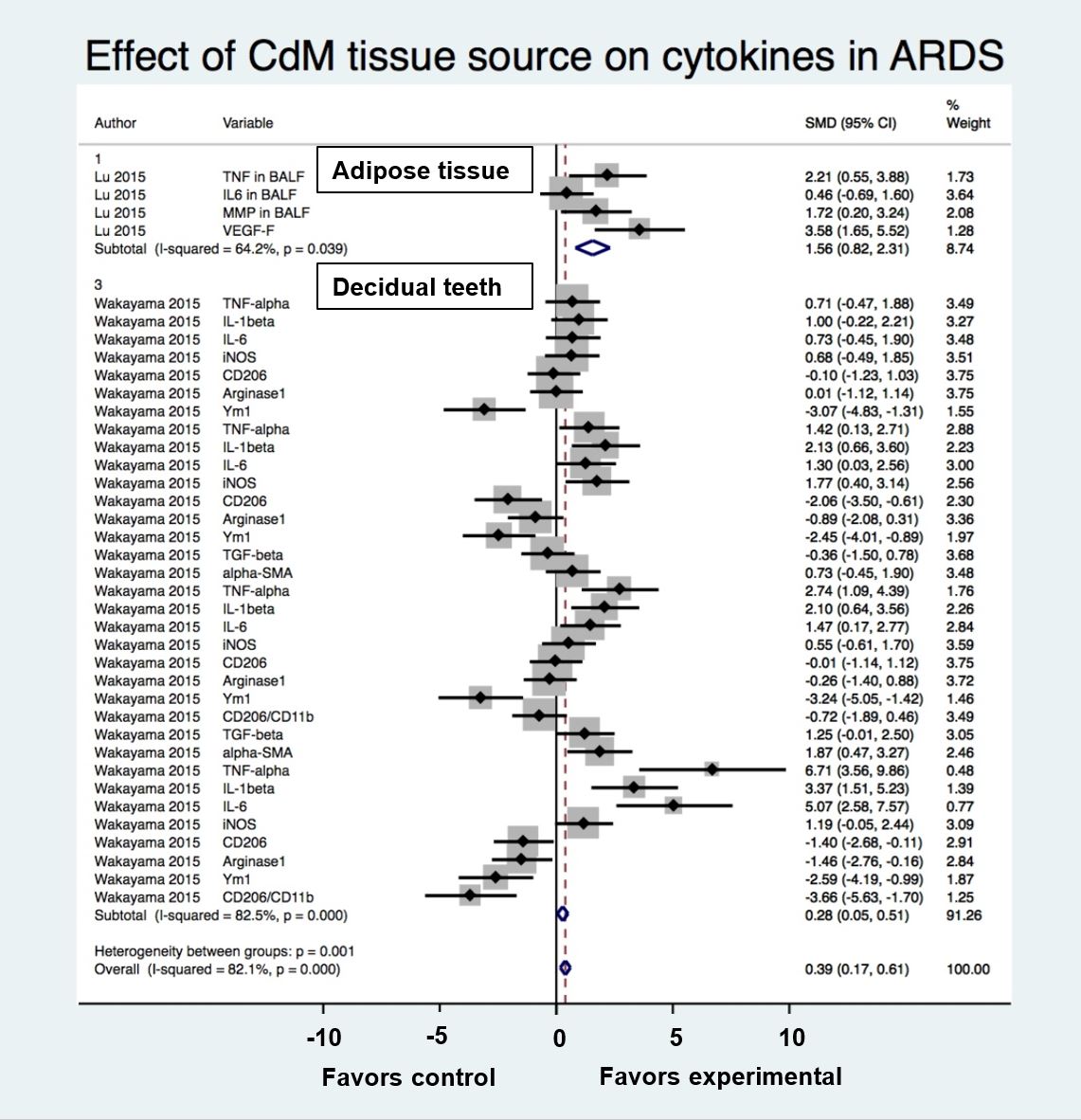

Supplement: Supplementary file 4 — Additional file 4: Figure S4. Effect size of CdM tissue source on inflammatory cytokines in ARDS. Forest plots demonstrating SMD and 95% CI. [file 12931_2019_1212_MOESM4_ESM.jpg]
