# Supplementary material for: Mesenchymal stromal cell conditioned media for lung disease: a systematic review and meta-analysis of preclinical studies
Source: Respir Res. 2019 Oct 30;20:239. doi: 10.1186/s12931-019-1212-x (PMC6822429; doi:10.1186/s12931-019-1212-x)
Supplement: Supplementary file 5 — Additional file 5: Table S1. SYRCLE 2014 protocol format. [file 12931_2019_1212_MOESM5_ESM.docx]

| 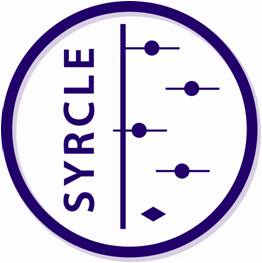 **Systematic Review Protocol for Animal Intervention Studies**  **Format by SYRCLE (**[**www.syrcle.nl**](http://www.syrcle.nl)**)**  **Version 2.0 (December 2014)** | | | | |
| --- | --- | --- | --- | --- |
| **Item #** | **Section/Subsection/Item** | **Description** | | **Check for approval** |
|  | A. General | | | |
| 1. | Title of the review | Effects of mesenchymal stem cell conditioned media on lung inflammation in animal models of pediatric lung disease: a systematic review | |  |
| 2. | Authors (names, affiliations, contributions) | **Evan Dittmar:** conception, study design, search, data collection, protocol writing, manuscript writing  **Chimobi Emukah:** conception, study design, search, data collection, protocol writing, manuscript writing  **Alvaro Moreira MD, MSc:** conception, study design, data collection and analysis, manuscript revision, supervision  University of Texas Health San Antonio  Department of Pediatrics, Division of Neonatology, | |  |
| 3. | Other contributors (names, affiliations, contributions) | None | |  |
| 4. | Contact person + e-mail address | **Alvaro Moreira**: MoreiraA@uthscsa.edu | |  |
| 5. | Funding sources/sponsors | National Center for Advancing Translational Sciences, National Institutes of Health, through Grant **KL2 TR001118.** This study was also supported by The University of Texas Health San Antonio School of Medicine Clinical Investigator Kickstart Pilot Grant. | |  |
| 6. | Conflicts of interest | None | |  |
| 7. | Date and location of protocol registration | CAMARADES | |  |
| 8. | Registration number (if applicable) | N/A | |  |
| 9. | Stage of review at time of registration | Preliminary searches | |  |
|  | B. Objectives | | | |
|  | Background | | | |
| 10. | What is already known about this disease/model/intervention? Why is it important to do this review? | Preclinical studies have now established that mesenchymal stem cell (MSC) therapy is a promising approach to treat multiple pediatric lung diseases. MSCs exert their therapeutic benefit by releasing biologic factors known to alleviate lung disease/injury. These trophic factors can be collectively gathered from the media where stem cells are cultured, collectively referred to as conditioned media (CdM). Animal studies demonstrate CdM may be as effective as MSCs in alleviating lung inflammation. Therefore, the purpose of this systematic review is to assess the efficacy of MSC CdM therapy in animal models of pediatric lung disease. | |  |
|  | Research question | | | |
| 11. | Specify the disease/health problem of interest | Pediatric lung diseases: acute respiratory distress, asthma, bronchopulmonary dysplasia, cystic fibrosis, pulmonary hypertension, pneumonia | |  |
| 12. | Specify the population/species studied | Animal models of pediatric lung disease (as listed above) | |  |
| 13. | Specify the intervention/exposure | Mesenchymal stem cell conditioned media | |  |
| 14. | Specify the control population | Placebo or no treatment | |  |
| 15. | Specify the outcome measures | Primary outcome: Inflammation  Secondary outcome: Lung architecture, Lung function | |  |
| 16. | State your research question (based on items 11-15) | What are the effects of mesenchymal stem cell conditioned media on experimental models of pediatric lung disease? | |  |
|  | C. Methods | | | |
|  | Search and study identification | | | |
| 17. | Identify literature databases to search (*e.g.* Pubmed, Embase, Web of science) | □xMEDLINE via PubMed □Web of Science  □xSCOPUS □EMBASE  □xOther, namely: Science Direct, CINAHL, Google Scholar  □Specific journal(s), namely: | |  |
| 18. | Define electronic search strategies (*e.g.* use the [step by step search guide^15^](http://www.ncbi.nlm.nih.gov/pmc/articles/PMC3265183/pdf/LA-11-087.pdf) and animal search filters[^20,^](http://www.ncbi.nlm.nih.gov/pmc/articles/PMC3104815/pdf/LA-09-117.pdf) [^21^](http://lan.sagepub.com/content/48/1/88.full.pdf+html)) |  | |  |
| 19. | Identify other sources for study identification | □xReference lists of included studies □Books  □xReference lists of relevant reviews  □Conference proceedings, namely:  □Contacting authors/ organisations, namely:  □Other, namely: | |  |
| 20. | Define search strategy for these other sources | Screening the reference lists for relevant titles and screening the abstracts of these relevant titles | |  |
|  | Study selection | | | |
| 21. | Define screening phases (*e.g.* pre-screening based on title/abstract, full text screening, both) | First phase: screening by title and abstract  Second phase: full text screening of eligible articles  Full text studies that do not meet inclusion will be incorporated into the flow diagram with reasons for exclusion | |  |
| 22. | Specify (a) the number of reviewers per screening phase and (b) how discrepancies will be resolved | a) Two investigators (E. Dittmar & C. Emukah) will independently screen all the abstracts/full texts for the inclusion criteria. b) Differences of opinion in either phase that cannot be resolved by discussion will be resolved by consulting a third investigator (A. Moreira). | |  |
|  | *Define all inclusion and exclusion criteria based on:* | | | |
| 23. | Type of study (design) | Inclusion criteria: pre-clinical studies  Exclusion criteria: non-intervention studies, no control group, co-intervention studies | |  |
| 24. | Type of animals/population (*e.g.* age, gender, disease model) | Inclusion criteria: animal models of pediatric lung disease, all genders  Exclusion criteria: humans, in-vitro, non-pediatric models of lung disease | |  |
| 25. | Type of intervention (*e.g.* dosage, timing, frequency) | Inclusion criteria: administration of MSC CdM- all dosages, timing, and frequency; MSCs can be derived from any tissue source  Exclusion criteria: CdM derived from other types of stem cells, CdM combined with other derivatives, sole use of exosomes/miRNA or other biologic factors found in CdM | |  |
| 26. | Outcome measures | Inclusion criteria: Primary-assessments of lung  Exclusion criteria: | |  |
| 27. | Language restrictions | Inclusion criteria: English and Spanish  Exclusion criteria: all other languages | |  |
| 28. | Publication date restrictions | Inclusion criteria: no publication date restrictions  Exclusion criteria: | |  |
| 29. | Other | Inclusion criteria:  Exclusion criteria: | |  |
| 30. | Sort and prioritize your exclusion criteria per selection phase | Selection phase: title and abstract screening  1. Not a primary study  2. Not an in vivo animal study  3. Not pediatric lung disease  4. No MSC CdM treatment  5. Adult animal  Selection phase: full text screening  1. Not a primary study  2. Not an in vivo animal study  3. Not pediatric lung disease  4. No MSC CdM treatment  5. No assessment of lung inflammation  5. No control group  6. Co-intervention studies | |  |
|  | Study characteristics to be extracted (for assessment of external validity, reporting quality) | | | |
| 31. | Study ID (*e.g.* authors, year) | Authors, journal, title, year, language, contact author e-mail | |  |
| 32. | Study design characteristics (*e.g.* experimental groups, number of animals) | Number of animals in experimental and control groups, reporting of randomization process, power calculation reported, method(s) to induce lung disease | |  |
| 33. | Animal model characteristics (*e.g.* species, gender, disease induction) | Animal species, strain, age, gender, weight, and immune status | |  |
| 34. | Intervention characteristics (*e.g.* intervention, timing, duration) | Source, dose, delivery, timing, and frequency of intervention | |  |
| 35. | Outcome measures | Assessments of lung inflammation | |  |
| 36. | Other (*e.g.* drop-outs) | Assessments of lung architecture, lung function, reason for exclusion | |  |
|  | Assessment risk of bias (internal validity) or study quality | | | |
| 37. | Specify (a) the number of reviewers assessing the risk of bias/study quality in each study and (b) how discrepancies will be resolved | a) Two investigators (E. Dittmar & C. Emukah) will independently screen all the abstracts/full texts for the inclusion criteria. b) Differences of opinion in either phase that cannot be resolved by discussion will be resolved by consulting a third investigator (A. Moreira) | |  |
| 38. | Define criteria to assess (a) the internal validity of included studies (*e.g.* selection, performance, detection and attrition bias) and/or (b) other study quality measures (*e.g.* reporting quality, power) | □xBy use of [SYRCLE's Risk of Bias tool^4^](http://www.biomedcentral.com/1471-2288/14/43/abstract)  □By use of SYRCLE’s Risk of Bias tool, adapted as follows:  □By use of [CAMARADES' study quality checklist, e.g ^22^](http://www.ncbi.nlm.nih.gov/pubmed/15060322)  □By use of CAMARADES' study quality checklist, adapted as follows:  □Other criteria, namely: | |  |
|  | Collection of outcome data | | | |
| 39. | For each outcome measure, define the type of data to be extracted (*e.g.* continuous/dichotomous, unit of measurement) | All outcome measures will be expressed through study units of measure, values expressed as continuous measures will be recorded as means +/- SD, SEM or median +/- IQR | |  |
| 40. | Methods for data extraction/retrieval (*e.g.* first extraction from graphs using a digital screen ruler, then contacting authors) | Extraction from text, tables, and figures (GetData graph digitizer 2.26)  Contact authors in case of missing data | |  |
| 41. | Specify (a) the number of reviewers extracting data and (b) how discrepancies will be resolved | a) Two investigators (E. Dittmar & C. Emukah) will independently screen all the abstracts/full texts for the inclusion criteria. b) Differences of opinion in either phase that cannot be resolved by discussion will be resolved by consulting a third investigator (A. Moreira) | |  |
|  | Data analysis/synthesis | | | |
| 42. | Specify (per outcome measure) how you are planning to combine/compare the data (*e.g.* descriptive summary, meta-analysis) | For sufficient data, we will conduct a meta-analysis for eligible studies. If insufficient data to measure outcomes, we will provide a descriptive summary of study results | |  |
| 43. | Specify (per outcome measure) how it will be decided whether a meta-analysis will be performed | A minimum of 4 articles for the same outcome is required. High heterogeneity is expected between studies due to differences in the study designs. We will preform a meta-regression analysis to investigate sources of heterogeneity. | |  |
|  | *If a meta-analysis seems feasible/sensible, specify (for each outcome measure):* | | | |
| 44. | The effect measure to be used (*e.g.* mean difference, standardized mean difference, risk ratio, odds ratio) | Continuous outcomes will be analysed using standardized mean differences (95% CI) | |  |
| 45. | The statistical model of analysis (*e.g.* random or fixed effects model) | Random effects model | |  |
| 46. | The statistical methods to assess heterogeneity (*e.g.* I^2^, Q) | I^2^ | |  |
| 47. | Which study characteristics will be examined as potential source of heterogeneity (subgroup analysis) | Study design: experimental and control groups, lung disease model, measures of inflammation, outcome time  Animal model: species, strain, age, gender  CdM: source, dose, delivery, timing, frequency, transplant method (allogeneic, xenogeneic, autologous, etc.) | |  |
| 48. | Any sensitivity analyses you propose to perform | If high heterogeneity is observed (≥70%), subgroup analyses will be conducted | |  |
| 49. | Other details meta-analysis (*e.g.* correction for multiple testing, correction for multiple use of control group) | N/A | |  |
| 50. | The method for assessment of publication bias | Funnel plot assessment  Egger’s regression | |  |
|  | | | | |
| Final approval by (names, affiliations): | | Evan Dittmar  Chimobi Emukah  Alvaro Moreira MD, MSc  UT Health San Antonio  Department of Pediatrics, Division of Neonatology  7703 Floyd Curl Drive MC 7812  San Antonio, TX, USA 78229 | Date: August 3^rd^, 2018 | |
