# Supplementary material for: Mesenchymal stromal cell conditioned media for lung disease: a systematic review and meta-analysis of preclinical studies
Source: Respir Res. 2019 Oct 30;20:239. doi: 10.1186/s12931-019-1212-x (PMC6822429; doi:10.1186/s12931-019-1212-x)
Supplement: Supplementary file 6 — Additional file 6: Table S2. Literature search terms. [file 12931_2019_1212_MOESM6_ESM.docx]

| Database | Terms Searched |
| --- | --- |
| Pubmed  (Search date: August 7, 2018) | (((((((((ANIMAL[MeSH Terms]) OR ANIMAL) OR PRECLINICAL) OR EXPERIMENTAL) )) AND Animals[Mesh:noexp])) AND ((((((((((((((((((((((((((PEDIATRIC LUNG DISEASE[MeSH Terms]) OR PEDIATRIC LUNG DISEASE) OR NEONATAL LUNG DISEASE) OR CHILDHOOD LUNG DISEASE) OR PEDIATRIC RESPIRATORY ILLNESS) OR BRONCHOPULMONARY DYSPLASIA) OR BPD) OR CHRONIC LUNG DISEASE) OR HYPEROXIA-INDUCED) OR ASTHMA) OR ALLERGIC REACTIVE DISEASE) OR HYPERREACTIVE LUNGS) OR PULMONARY HYPERTENSION) OR PAH) OR RIGHT VENTRICLE FAILURE) OR ACUTE RESPIRATORY DISTRESS SYNDROME) OR ARDS) OR ACUTE RESPIRATORY FAILURE) OR CYSTIC FIBROSIS) OR CF) OR PNEUMONIA) OR PNA) OR COMMUNITY ACQUIRED LUNG DISEASE) )) AND Animals[Mesh:noexp])) AND ((((((((((mesenchymal STEM CELL CONDITIONED MEDIA[MeSH Terms]) OR mesenchymal STEM CELL CONDITIONED MEDIA) OR MSC MEDIA) OR STROMAL CELL MEDIA) OR CONDITIONED MEDIA) OR PARACRINE FACTORS) OR CELL FREE PRODUCT) )) AND Animals[Mesh:noexp]) |
| Scopus  (Search date: August 14, 2018) | (animal  OR  preclinical  OR  experimental )  AND  ( pediatric  AND lung  AND disease  OR  neonatal  AND lung  AND disease )  AND  ( mesenchymal  AND stem  AND cell  AND conditioned  AND media  OR  conditioned  AND media  OR  paracrine  AND factors  OR  cell-free  AND product ) ​ |
| Science Direct  (Search date: September 5, 2018) | pediatric lung disease, stem cell conditioned media, animal |
| Google Scholar  (Search date: September 5, 2018) | pediatric lung disease, stem cell conditioned media, animal |
| CINAHL  (Search date: September 5, 2018) | (MH “Stem Cells”) OR (MH “Stem Cell Research”) OR “stem cell AND lung disease AND animal” |

**Supplementary Table 2.** Literature search terms
