# Supplementary material for: Mesenchymal stromal cell conditioned media for lung disease: a systematic review and meta-analysis of preclinical studies
Source: Respir Res. 2019 Oct 30;20:239. doi: 10.1186/s12931-019-1212-x (PMC6822429; doi:10.1186/s12931-019-1212-x)
Supplement: Supplementary file 7 — Additional file 7: Table S3. Number of animals in studies. [file 12931_2019_1212_MOESM7_ESM.docx]

**Supplementary Table 3.** Number of animals in study

| **Sample characteristics** | **N** |
| --- | --- |
| Number of papers | 10 |
| Number of pulmonary diseases | 4 |
| Number of animals with pulmonary disease | 62 |
| Number of animals with pulmonary disease treated with CdM | 68 |
| Number of animals with pulmonary disease treated with MSCs | 52 |
| Total number of animals | 182 |
| Median number of animals in pulmonary disease group | 6 |
| Median number of animals in pulmonary disease group treated with CdM | 6 |
| Median number of animals in pulmonary disease group treated with MSCs | 6 |

**Abbreviations:** Conditioned media (CdM); Mesenchymal stromal cells (MSCs)
