# Supplementary material for: Mesenchymal stromal cell conditioned media for lung disease: a systematic review and meta-analysis of preclinical studies
Source: Respir Res. 2019 Oct 30;20:239. doi: 10.1186/s12931-019-1212-x (PMC6822429; doi:10.1186/s12931-019-1212-x)
Supplement: Supplementary file 8 — Additional file 8: Table S4. CdM characteristics. [file 12931_2019_1212_MOESM8_ESM.docx]

| **Author (Year)** | **Source** | **Were MSCs purchased or self-isolated?** | **Cell expansion media** | **Incubation time** | **Passage number** | **Dose** | **Notes** |
| --- | --- | --- | --- | --- | --- | --- | --- |
| **Ahmadi (2016)** | Rat bone marrow MSCs | Self-isolated | DMEM | 72 hours | 3 | 50 µl | Concentrated 50x; MW cutoff 4 kDa |
| **Ahmadi (2017)** | Rat bone marrow MSCs | Self-isolated | αMEM | 72 hours | 3 | 50 µl | Concentrated 50x; MW cutoff 4 kDa; concentration 500µg/mL |
| **Aslam (2009)** | Mouse bone marrow MSCs | Self-isolated | DMEM | 24 hours | 7-10 | 50 µl | Concentrated 10x; MW cutoff 10 kDa |
| **Cruz (2015)** | Mouse bone marrow MSCs | Texas A&M stem cell core | IMDM | 24 hours | <7 | 200 µl | Concentrated 25x; MW cutoff 3 kDa |
| **Ionescu (2012)** | Mouse bone marrow MSCs | Self-isolated | DMEM | 24 hours | 2-8 | 30 µl | Concentrated 25x; MW cutoff 3 kDa |
| **Lee (2012)** | Mouse bone marrow MSCs | Self-isolated | αMEM | 24 hours | 3-4 | 50 µl | Concentrated to protein [0.1-0.5 mg/mL] |
| **Lu (2012)** | Human adipose MSCs | Self-isolated | EBM2 | 24 hours | 3 | 200 µl | Concentrated 10x; MW cutoff 3 kDa |
| **Rathinasabapathy (2012)** | Rat adipose MSCs | Self-isolated | DMEM | 24 hours | 2-5 | 100 µl | Concentrated to dose; MW cutoff 3 kDa |
| **Sutsko (2012)** | Rat bone marrow MSCs | Self-isolated | αMEM | 24 hours | NR | 50 µl | NR |
| **Wakayama (2015)** | Human teeth MSCs | Self-isolated | DMEM | 48 hours | 3-9 | 500 µl | Average protein [3 µg/mL] |

**Supplementary Table 4.** Conditioned media characteristics

(DMEM) Dulbecco’s Modified Eagle Medium, (IMDM) Iscove’s Modified Dulbecco’s Medium, (MSCs) Mesenchymal stem cells; (MW) Molecular weight; (NR) Not reported
