# Supplementary material for: Mesenchymal stromal cell conditioned media for lung disease: a systematic review and meta-analysis of preclinical studies
Source: Respir Res. 2019 Oct 30;20:239. doi: 10.1186/s12931-019-1212-x (PMC6822429; doi:10.1186/s12931-019-1212-x)
Supplement: Supplementary file 9 — Additional file 9: Table S5. Current clinical trials in asthma, BPD, ARDS, PH. [file 12931_2019_1212_MOESM9_ESM.docx]

| **Sponsor (NCT)** | **Disease** | **Methods** | **Participants** | **Interventions** | **Comparison** | **Outcome** | **Notes** |
| --- | --- | --- | --- | --- | --- | --- | --- |
| **University of Miami (03137199)** | Asthma | Phase I study comparing two doses: 20M (n=3) vs 100M (n=3) | Adults 18-65 years; n=6 | Allogeneic human bone marrow MSCs (IV) | NA | Safety | Recruiting; Assessing adverse events, pulmonary parameters (DLCO, PFT), 6MWT, QOL, and laboratory tests |
| **Translational Biosciences**  **(02192736)** | Asthma | Phase I/II, treatment given once/week for one month | Adults 21-65 years; n=20 | Allogeneic human umbilical cord tissue-derived MSC trophic factors (IN) | NA | Safety and efficacy | Active, not recruiting; Examining adverse events, PFT, QOL |
| **University of California-San Francisco**  **(01775774)** | ALI/ARDS | Phase I, open-label, dose escalation (1M, 5M, 10M cells/kg), multi-center trial | Adults 18+ years; Three subjects per group; n=9 | Allogeneic human bone marrow MSCs | NA | Safety | Completed; No treatment-related adverse events; 2 deaths and 1 with multiple emboli not deemed secondary to MSCs; cells may improve LIS, organ failure, markers of inflammation/injury |
| **Sun Yat-sen University (03608592)** | ALI/ARDS | 1M cells/kg mixed with normal saline will be infused | Adults 18+ years; n=26 | Allogeneic human umbilical cord-derived MSCs (IV) | NA | Safety | Recruiting; Evaluating adverse events, ventilation free days, OI, LIS, ventilator parameters, and serum/tracheal biomarkers |
| **MD Anderson Cancer Center**  **(02804945)** | ALI/ARDS in pts with malignancy | Phase II; single infusion of 3M cells/kg | Adults 18+; n=20 | Allogeneic human bone marrow MSCs (IV) | NA | Safety | Completed; no publication/summary of findings posted |
| **Affiliated Hospital to Academy of Military Medical Sciences (02444455)** | ALI | Phase I/II, open-label infusion of 500K cells/kg; total of 3 doses | Adult 35-70 years; n=20 | Allogeneic human umbilical cord-derived MSCs (IV) | NA | Safety and efficacy | Unknown/recruiting status; Examining adverse events, pulmonary function by chest imaging, serum biomarkers and blood gas analysis |
| **Shaoxing Second Hospital**  **(01902082)** | ARDS | Phase I, 1M cells/kg (n=6), compared to placebo (n=6) | Adult 18-90 years; n=12 | Allogeneic human adipose-derived MSCs (IV) | Normal saline | Safety | Completed; no infusion toxicities; hospital days were similar between placebo and MSC group; similar liver and kidney function; serum surfactant protein D levels were lower in the MSC group but not statistically different from placebo |
| **Asian Medical Center (02112500)** | ARDS | Phase II | Adult 20-80 years; n=10 | Autologous human bone marrow MSCs (IV) | NA | Efficacy | Unknown recruiting status; primary outcome is OI; lung, hemodynamic parameters, cytokines, inflammation, mortality |
| **Belfast Health and Social Care Trust** | ARDS | Phase I/II, open-label, dose-escalation | Adolescent and adult 16+ years; n=75 | Allogeneic human umbilical cord-derived CD362 enriched MSCs (IV) | Plasmalyte | Safety and efficacy | Recruiting; OI and adverse events are primary outcomes; also collecting pulmonary measures |
| **S-Evans Biosciences Co (02095444)** | ALI/ARDS | Phase I/II, 10M cells/kg with 4 administrations over 2 weeks | Adults > 20 years; n=20 | Allogeneic menstrual blood stem cells  (IV) | NA | Safety and efficacy | Unknown recruiting status; primarily assessing lung injury |
| **Liaocheng People’s Hospital**  **(04055415)** | PH | Phase I/II, 10M cells/kg with 2 administrations over 2 weeks | Adults 40-75 years; n=60 | Allogeneic human adipose derived MSCs (IV) | Conventional therapy | Safety and efficacy | Recruiting, pulmonary vascular resistance, QOL, serum markers, 6MWD |
| **Cedars-Sinai Medical Center**  **(03145298)** | PH | Phase Ia-6 pts will receive 50M or 100M cells; Phase 1b-double-blind, randomized, placebo-controlled study in 20 pts | Adults 40-75 years; n=26 | Allogeneic human cardiosphere-derived stem cells (IV) | Placebo | Safety | Recruiting, early and long-term measures of safety (gas exchange, hemodynamics, arrhythmias, death, hospitalization, ventricular failure); exploratory measures of RV function |
| **Zhejiang University**  **(00641836)** | PH | Phase II, Prospective, randomized trial, mean administration of 11M cells | Adults 18-60 years; n=31 (16 received cells) | Autologous endothelial progenitor cells (IV) | Conventional therapy | Safety and efficacy | Completed; 6MWD improved with cell therapy; no severe adverse events with infusion |
| **Northern Therapeutics**  **(00469027)** | PH | Phase I Dose-escalating (7M, 23M, and 50M) | Adults 18-80 years; n=7 | Autologous endothelial nitric oxide synthase-transfected progenitor cells (PAC) | NA | Safety | Completed; no evidence of hemodynamic instability with infusion of cells; 1 death at discharge possibly related to cell therapy; 1 patient with sepsis at 9 months after cell delivery deemed unrelated to cell product; improvement in 6MWD |
| **Northern Therapeutics**  **(03001414)** | PH | Phase II, randomized, double-blind, placebo-controlled, 3-arm protocol; 4 monthly infusions  Arm 1: placebo (6 mo), endothelial progenitor cells (EPCs, 6 mo), 80M total cells  Arm 2: EPCs (6 mo), placebo (6 mo), 80M total cells  Arm 3: EPCs (6 mo), EPCs (6 mo), 160M total cells | Adults 18-80 years; n =45 | Autologous endothelial nitric oxide synthase-transfected progenitor cells (IV) | Placebo | Safety and efficacy | Recruiting; primary outcome-6MWD from baseline at 6 months; 6MWD at different timepoints, pulmonary vascular resistance, death or clinical worsening, change in RV function, QOL |
| **Children’s Hospital of Fudan University**  **(03645525)** | BPD | Phase I/II, 20M cell/kg | Preterm neonates up to 3 weeks; n =180 | Allogeneic human umbilical cord-derived MSCs (IT) | Normal saline | Safety and efficacy | Not yet recruiting; primary outcome is oxygen requirement 3 days after cell transplantation, secondary measures include oxygen % at 7 days, ventilator days, incidence and severity of BPD, survival, growth velocity, BAL cytokine, chest xray, and vitals |
| **Children’s Hospital of Chongqing Medical University**  **(03558334)** | BPD | Phase I, 1M vs. 5M cells/kg | Preterm neonates with moderate/severed BPD; n =12 | Allogeneic human umbilical cord-derived MSCs (IV) | NA | Safety | Recruiting, assessment of adverse reactions with secondary measures including imaging, vitals, and growth |
| **Vinmec Research Institute of Stem Cell and Gene Technology**  **(04062136)** | BPD | Phase I, 1M cells/kg at baseline and one week later | Preterm neonate 1mo-6 mo; n=10 | Allogeneic human umbilical cord-derived MSCs (IV) | NA | Safety | Recruiting, primary outcome includes adverse events, secondary outcome-lung fibrosis by chest CT |
| **Children’s Hospital of Chongqing Medical University**  **(03601416)** | BPD | Phase II, 1M vs. 5M cells/kg | Up to 1 year; n=57 | Allogeneic human umbilical cord-derived MSCs (IV) | NA | Efficacy | Not yet recruiting; examining duration of oxygen therapy; secondary measures-vitals; exploratory studies-chest CT |
| **Children’s Hospital of Chongqing Medical University**  **(03873506)** | BPD | Phase 1 follow up study for 1M vs. 5M cells/kg trial, assessments at 1, 3, 6, 12, and 24 months post cell treatment | 1-5-month old neonates; n=30 | Allogeneic human umbilical cord-derived MSCs (IV) | NA | Safety | Recruiting; Primary outcome-readmission rate and duration of hospital stay; secondary measures-survival, growth, tumorigenicity, developmental delay, blindness/deafness |
| **Fundaction para la Investigacion Biomedica del Hospital Universitario Ramon y Cajal**  **(02443961)** | BPD | Phase I, three doses of 5M cells | Preterm neonates <29 weeks and birthweight <1250 g; n=10 | Specifics not provided | NA | Safety | Not yet recruiting, evaluating safety and feasibility, other measures of interest include biomarker analysis, changes in echocardiographic findings and incidence of BPD/PH |
| **Daping Hospital and the Research Institute of Surgery of the Third Military Medical University**  **(03378063)** | BPD | Phase I | 1-3 mo old neonates; n=100 | Allogeneic human umbilical cord blood-derived MSCs (Unknown) | NA | Safety and efficacy | Recruiting, primary outcome is death with developmental testing as the secondary outcome |
| **Children’s Hospital of Chongqing Medical University**  **(03774537)** | BPD | Phase I/II, 1M vs. 5M cells/kg | Neonates between 3 to 14 days; n=20 | Allogeneic human umbilical cord-derived MSCs (IV) | NA | Safety and efficacy | Recruiting, assessment of adverse reactions with secondary measures including incidence and severity of BPD, imaging, vitals, and growth |
| **United Therapeutics**  **(03857841)** | BPD | Phase I, 20 vs. 60 vs. 200 pmol phospholipid/kg | Neonates between 3 to 14 days; n=18 | Allogeneic human bone marrow-derived MSC extracellular vesicles (IV) | Phosphate buffered saline | Safety | Recruiting, safety as primary outcome, secondary outcomes include incidence and severity of BPD, death at 36 weeks post-menstrual age, duration of hospitalization and mechanical ventilation, duration of supplemental oxygen, respiratory severity score |
| **China Medical University Hospital**  **(01207869)** | BPD | Phase I, 3M cell/kg | Neonates up to 6 months of age; n=10 | Allogeneic human umbilical cord-derived MSCs (IT) | Normal saline | Safety | Unknown recruitment status, primary outcome-cytokine measurements of BAL; secondary outcome-BPD severity on chest xray |
| **Guangdong Women and Children Hospital**  **(03683953)** | BPD | Phase I, 25M cells/kg | >28-week gestation neonates with > 2 weeks of mechanical ventilation; n=200 | MSCs-source not mentioned (IT) | Normal saline | Safety | Not yet recruiting, primary outcome-incidence of BPD |
| **Meridigen Biotech Co, Ltd**  **(03631420)** | BPD | Phase I, dose-escalation: 3M vs 10M vs. 30M cells/kg | Born at <28 weeks and birthweight < 1250 g and diagnosed with severe BPD; n=9 | Allogeneic, human umbilical cord-derived MSCs (not mentioned) | NA | Safety | Not yet recruiting, incidence and frequency of adverse events; secondary-death, oxygen saturation, xray changes, inflammatory markers, duration of oxygen and mechanical ventilation |
| **Medipost Co Ltd**  **(01297205)** | BPD | Phase I, dose-escalation, 10M cells/kg (n=3), 20M cells/kg (n=6) | 23-29-week gestation neonates; n=9 | Allogeneic human umbilical cord blood-derived MSCs (IT) | NA | Safety | Completed; no serious adverse effects reported in all doses; reduction in inflammatory markers in tracheal aspirate |
| **Medipost Co Ltd**  **(01828957)** | BPD | Phase II, 10M cells/kg | ≥23 weeks and <29 weeks with a birthweight between 500 and 1250 g; n=70 | Allogeneic human umbilical cord blood-derived MSCs (IT) | Normal saline | Efficacy | Completed, no results posted to date; primary-incidence of BPD or mortality at 36 weeks post-menstrual age; secondary-ventilator days, incidence of BPD, duration of oxygen use and positive pressure ventilation, growth, adverse events, other neonatal morbidities |
| **Medipost Co Ltd**  **(04003857)** | BPD | Phase II, follow-up study at timepoints-6, 12,18, and 24 months corrected age, and 36, 48, and 60 months after birth | ≥23 weeks and <29 weeks with a birthweight between 500 and 1250 g; n=60 | Allogeneic human umbilical cord blood-derived MSCs (IT) | Normal saline | Efficacy | Recruiting, primary outcome includes number of hospitalizations at different timepoints; secondary measures include mortality, growth, development, ER visits, deafness, blindness, respiratory medications |
| **Medipost Co Ltd**  **(01897987)** | BPD | Phase II, follow-up study at timepoints-6, 12,18, 24,36, 48, and 60 months corrected age | ≥23 weeks and <29 weeks with a birthweight between 500 g and 1250 g; n=70 | Allogeneic human umbilical cord blood-derived MSCs (IT) | Normal saline | Efficacy | Recruiting, primary outcome includes readmission rate and duration of hospital stay due to respiratory infection at all timepoints; secondary measures include respiratory medications, survival, growth, neurodevelopment, ER visits, deafness, blindness, and changes in vital signs/physical exam |
| **Medipost America Inc**  **(02381366)** | BPD | Phase I/II, 10M vs 20M cells/kg, n=6/dose | ≥23 weeks and <28 weeks with a birthweight between 500 g and 1000 g; n=12 | Allogeneic human umbilical cord blood-derived MSCs (IT) | NA | Safety and efficacy | Completed; no adverse events were related to study drug; 10 of 12 neonates developed severe BPD |
| **Medipost Co Ltd**  **(02023788)** | BPD | Phase I long-term follow up, 10M (n=3) vs 20M (n=6) cells/kg | 45 mo to 63 mofollo-up study after administration of cells; n=9 | Allogeneic human umbilical cord blood-derived MSCs (IT) | NA | Safety | Completed; 8 of 9 patients survived; death in patient secondary to sepsis at 6 months; MSC group with lower incidence of home oxygen use and higher bodyweight; no infant diagnosed with CP, blindness, or developmental delay |

6-minute walk test/distance (6MWT/D); Acute lung injury, acute respiratory distress syndrome (ALI/ARDS); Bronchoalveolar lavage (BAL); Bronchopulmonary dysplasia (BPD); Cerebral palsy (CP); Diffusing capacity (DLCO); Endothelial progenitor cell (EPC); Intranasal (IN); Intratracheal (IT); Intravenous (IV); Lung injury score (LIS); Million (M); Not applicable (NA); Oxygenation index (OI); Patients (pts); Pulmonary artery catheter (PAC); Pulmonary function test (PFT); Pulmonary hypertension (PH); Quality of life (QOL); Right ventricle (RV); Thousand (K).
