# Supplementary material for: Mesenchymal stromal cell conditioned media for lung disease: a systematic review and meta-analysis of preclinical studies
Source: Respir Res. 2019 Oct 30;20:239. doi: 10.1186/s12931-019-1212-x (PMC6822429; doi:10.1186/s12931-019-1212-x)
Supplement: Supplementary file 10 — Additional file 10: Table S6. Advantages of CdM vs. MSCs. [file 12931_2019_1212_MOESM10_ESM.docx]

| Advantages of **CdM** | Advantages of **MSCs** |
| --- | --- |
| Safety: decreased chance for tumorigenicity or cell rejection | Longer half-life/survival |
| Similar inflammatory effects of MSCs | Cell-cell interaction with injured cell |
| Contains biologically active agents found in MSCs | Ability to adapt to microenvironment |
| Stem cell potency assays typically involve analysis of secreted factors into the media | More experience with their use in preclinical and clinical studies |
| Low risk for thrombosis | Ease of tracking cells |
| Scalability |  |
| Media can be stored without freezing and therefore no concerns for cell viability between freeze-thaw cycles |  |

**Supplementary Table 6. CdM vs. MSCs**
